# Phytochemical Study and In Vitro Antioxidant Activity of *Helianthemum cinereum* Along with Antitumor Activity of the Isolated *trans*-Tiliroside and Luteolin 4′-*O*-β-Xyloside

**DOI:** 10.3390/molecules29245935

**Published:** 2024-12-16

**Authors:** Anis Bertella, Abla Smadi, Hakim Benhabrou, Diana Salvador, Magdalena Wrona, Helena Oliveira, Abouamama Sidaoui, Georgiana Gavril-Luminita, Diana C. G. A. Pinto, Ewa Olewnik-Kruszkowska, Cristina Nerín, Artur M. S. Silva, Fatma Bitam

**Affiliations:** 1 Department of Molecular and Cellular Biology, Faculty of Life and Nature Sciences, Abbes Laghrour University Khenchela, BP 1252 Road of Batna, Khenchela 40004, Algeria; 2 Laboratory of Chemistry and Environmental Chemistry (LCCE), Department of Chemistry, Faculty of Matter Sciences, University of Batna 1, Batna 05000, Algeria; abla.smadi@univ-batna.dz (A.S.); hakim.benhabrou@univ-batna.dz (H.B.); 3 Department of Biology & CESAM, University of Aveiro, 3810-193 Aveiro, Portugal; diana.s@ua.pt (D.S.); holiveira@ua.pt (H.O.); 4 Institute of Bio- and Geosciences 2, Forschungszentrum Jülich GmbH, 52428 Jülich, Germany; m.wrona@fz-juelich.de; 5 Department of Biology, Faculty of Sciences and Technology, Amine Elokkal El Hadj Moussa Egakhamouk University of Tamanghasset, Tamanghasset 11000, Algeria; sidaouibouamama@univ-tam.dz; 6 Department of Bioinformatics, National Institute of Research and Development for Biological Sciences, 296 Splaiul Independentei, Sector 6, 060031 Bucharest, Romania; georgi.gavril@yahoo.com; 7 LAQV/REQUIMTE, Department of Chemistry, Campus Universitário de Santiago, University of Aveiro, 3810-193 Aveiro, Portugal; diana@ua.pt (D.C.G.A.P.); artur.silva@ua.pt (A.M.S.S.); 8 Physical Chemistry and Physicochemistry of Polymers, Faculty of Chemistry, Nicolaus Copernicus University in Torún, Gagarin 7 Street, 87-100 Torún, Poland; olewnik@umk.pl; 9 Departmento de Química Analítica, Instituto de Investigación en Ingeniería de Aragón (I3A), Escuela de Ingeniería y Arquitectura (EINA), Universidad de Zaragoza, María de Luna 3 (Edificio Torres Quevedo), 50018 Zaragoza, Spain; cnerin@unizar.es; 10 Department of Pharmacy, Faculty of Medicine, University of Batna 2, Batna 05000, Algeria

**Keywords:** Cistaceae, *Helianthemum*, NMR, flavonoids, antitumor activity, antioxidant activity

## Abstract

Twelve compounds (**1**–**12**), kaempferol (**1**), luteolin (**2**), luteolin 4′-*O-β*-xyloside (**3**), luteolin 4′-*O*-β-glucoside (**4**), quercetin 4′-*O*-β-xyloside (**5**), kaempferol-3-*O*-[6″-*O*-(E)-p-coumaroyl]-β-D-glucoside (*trans*-tiliroside) (**6**), protocatechuic acid (**7**), gallic acid (**8**), methyl gallate (**9**), ethyl gallate (**10**), shikimic acid-3-*O*-gallate (**11**), and 3,3′,4′-tri-*O*-methyl-ellagic acid 4-sulfate (**12**), were isolated and identified from the aerial parts of *Helianthemum cinereum* (Cav.) Pers (synonym: *Helianthemum rubellum* C. Presl. All compounds were isolated by applying different chromatographic procedures, such as silica gel, RP-18 and Sephadex LH-20 columns. The structures were elucidated by extensive spectroscopic methods, mainly nuclear magnetic resonance NMR 1D and 2D, and mass spectrometry, as well as by comparison with the reported spectroscopic data. The two organic extracts, ethyl acetate (EtOAc) and butanol (BuOH), were evaluated for their potent phenolic and flavonoid contents using the Folin–Ciocalteu and aluminum chloride colorimetric methods. Furthermore, the antioxidant activity of the two extracts was determined using the DPPH, FRAP, and ABTS methods. Pure *trans*-tiliroside (**6**), the main isolated compound, and luteolin 4′-*O*-β-xyloside (**3**) were evaluated for their antitumor activity against the lung cancer (A549), melanoma (A375) and pancreatic cancer (Mia PaCa-2 and Panc-1) cell lines by MTT assay.

## 1. Introduction

The Cistaceae family consists of over 200 species in eight genera (*Cistus*, *Fumana*, *Halimium*, *Helianthemum*, *Tuberaria*, *Crocanthemum*, *Hudsonia* and *Lechea*), mostly found in temperate and subtropical regions of the northern hemisphere, especially the western Mediterranean region, with a secondary center in the eastern United States [1]. The genus *Helianthemum* is the largest genus in the Cistaceae family, with more than 140 spread species [2,3]. Plants of the genus are frequently used in traditional medicine for their various therapeutic properties: healing, gastro-protective, anti-hemorrhoidal, antiseptic, antifungal, anti-inflammatory, analgesic, and antidiarrheal [4,5,6,7,8].

The leaves of *H. cinereum*, *H. apenninum*, *H. marifolium* and *H. syriacum* species are used for burns, wound healing, abdominal pain, diarrhea and gastroenteritis. Whole plants of this genus are ingested in decoctions and infusions for gastrointestinal problems. They are also applied to infected wounds or burns as a poultice [9]. In Algerian flora, this genus is presented by more than 40 species, most of them are generally found in western Algeria [10].

The phytochemistry of the genus *Helianthemum* has been reported recently by Mouffouk et al. [11]. In their minireview, the authors describe the results of the chemical investigations on 21 *Helianthemum* taxa that are described all over the world. According to these studies, secondary metabolites such as flavonoids, lignans, phenolic acids and sterols are the major constituents. Regarding the Algerian’s flora species: *H. lippii*, *H. sessiliflorum*, *H. Kahiricum*, *H. rifocomum*, *H. gelatum*, and *H. hirtum* are the only species that have been investigated [12,13,14,15,16,17,18]. A careful analysis demonstrated that flavones derived from luteolin and flavanols derived from quercetin and kaempferol are the main metabolites in the studied species. In addition, simple phenolics are also present and dominated by gallate derivatives and phenolic acids.

As part of our ongoing phytochemical research on Algerian *Helianthemum* species, we examined the ethyl acetate (EtOAc) and butanol (BuOH) extracts obtained from the aerial parts of *Helianthemum cinereum* (Cav.) Pers. (synonym: *Helianthemum rubellum* C. Presl).

To the best of our knowledge, no advanced chemical analysis has been reported on *H. cinereum* species and only screening and quantization of polyphenols of eleven taxa in south-eastern Spain have been performed by Rubio-Moraga et al. [9].

According to this study, polyphenols of *H. cinereum* were quantized from the methanolic and water extracts and luteolin, kaempferol were the most dominated compounds. In addition, the antioxidant and antimicrobial activities of these two extracts have been assessed [9].

Our chemical investigation on the Algerian *H. cinereum* species has revealed a secondary metabolite pattern dominated by flavonoids and phenolic compounds. Twelve compounds (**1**–**12**) were fully purified and characterized mainly by analyzing the NMR 1D and 2D spectra, as well as by comparing them with data reported in the literature. Additionally, the total phenolic and flavonoid contents and antioxidant activity were assessed on the ethyl acetate and butanol extracts. The results revealed that ethyl acetate extract shows a high amount of phenolic and flavonoid compounds and exhibits high antioxidant capacity. The main compound *trans*-tiliroside (**6**) and luteolin 4′-*O-β*-xyloside (**3**) have been tested in vitro for their antitumoral activity against the lung cancer (A549), melanoma (A375), pancreatic cancer (Mia PaCa-2 and Panc-1) and immortalized human keratinocytes (HaCaT) cell lines by MTT assay, and cell cycle analysis. These cell lines were selected based on the available literature, which highlights gaps in the understanding of the antitumor activity of *trans*-tiliroside on these cell lines. Furthermore, no previous studies have investigated the antitumor activity of luteolin 4′-*O*-β-xyloside, a rare flavonoid found in plants.

## 2. Results and Discussions

### 2.1. Phytochemical Study

**Figure 1:**
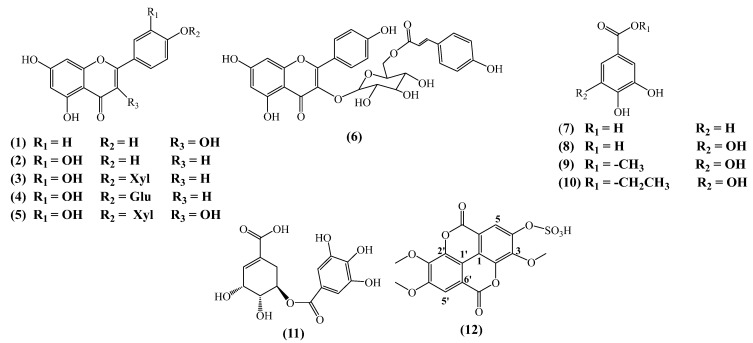
Chemical structure of isolated compounds from the aerial parts of *Helianthemum cinereum*.

The chemical composition of ethyl acetate (EtOAc) and butanol (BuOH) organic extracts of the aerial parts of *Helianthemum cinereum* is compared by TLC in different eluent systems. The composition and the relative distribution of the metabolites were characterized by a plethora of UV–visible metabolites in both extracts. However, a rich metabolite pattern was observed in the ethyl acetate extract—it showed less polar compounds in comparison with the butanol extract. Thus, both extracts were subjected to a series of chemical purifications starting by silica gel columns chromatography as a first step followed by Sephadex LH-20 columns and ending with C18-SPE cartridges. After separation (described in experimental part), nine compounds were identified (NMR and Mass spectrums in supplementary materials) from the ethyl acetate extract and three compounds from the butanol extract (Figure 1): kaempferol (**1**) [19], luteolin (**2**) [20], luteolin 4′-*O*-β-xyloside (**3**) [21], luteolin 4′-*O*-β-glucoside (**4**) [22], quercetin 4′-*O*-β-xyloside (**5**) [23], kaempferol-3-*O*-[6″-*O*-(E)-p-coumaroyl]-β-D-glucoside (*trans*-tiliroside) (**6**) [13,18,24,25], protocatechuic acid (**7**) [26], gallic acid (**8**) [16], methyl gallate (**9**) [27], ethyl gallate (**10**) [28], shikimic acid 3-*O*-gallate (**11**) [29], and 3,3′,4′-tri-*O*-methyl-ellagic acid 4-sulfate (**12**) [30,31].

To the best of our knowledge, compounds luteolin 4′-*O*-β-xyloside (**3**), luteolin 4′-*O*-β-glucoside (**4**), quercetin 4′-*O*-β-xyloside (**5**), ethyl gallate (phyllemblin) (**10**), shikimic acid 3-*O*-gallate (**11**), and 3,3′,4′-tri-O-methyl-ellagic acid 4-sulfate (**12**) are isolated here for the first time in the Cistaceae family. Luteolin 4′-*O*-β-xyloside (**3**), luteolin 4′-*O*-β-glucoside (**4**), and quercetin 4′-*O*-β-xyloside (**5**) are uncommon flavonoid 4′-*O*-β-glycosides, with limited data available in the literature [21,22,23]. Additionally, kaempferol (**1**) and luteolin (**2**) are reported from some species of the *Cistus* genus [20,32]. However, the other compounds (**6**), (**7**), (**8**) and (**9**) are reported from the genus *Helianthemum*, in particular, from the Algerian species: *H. lippii*, *H. sessiliflorum*, *H. Kahiricum*, *H. rifocomum*, *H. gelatum* and *H. hirtum* [12,13,14,15,16,17,18].

It is very interesting to mention here that we have isolated ellagic acid compound (**12**) as a sulphate derivative. The occurrence of ellagic acid derivatives is very common in the Cistaceae family [33], and their identification here is further supported by the occurrence of quercetin 3-sulphate and isorhamnetin 3-sulphate in the species *Helianthemum squamatum* [33,34,35]. Regarding their pharmacological effects on the human body, sulphated phenolics have demonstrated various biological activities, including anticoagulant, antiviral, antitumor, antibacterial, and anti-inflammatory properties. Compound (**12**) in turn, is reported to have a cytotoxic effect [31]. The finding of some of these compounds for the first time in the Cistaceae family highlights the significance of nature as a significant source of new compounds that continue to aid in a new approach and new avenues in plant chemistry, taxonomy and pharmacology. Flavonoid glycosides have been shown to exert diverse pharmacological activities. Some previous findings highlight the medicinal benefits of luteolin and some of its derivatives, particularly, their anticancer, anti-inflammatory, and antioxidant properties. Luteolin 4′-*O*-glucoside was identified as an IL-5 inhibitor from *Kummerowia striata* [36]; however, luteolin 4′-*O*-xyloside is reported as an enzymatic glycodiversification [31]. All these constatations, suggest that *Helianthemum cinereum* from the Cistaceae family is a valuable source of secondary metabolites, could hold potential for further research into this class of compounds, focusing on both their activities and taxonomy significance.

### 2.2. Total Phenolic and Flavonoids Contents

It is known that phenolic compounds are very important secondary metabolites of plants with redox properties responsible for antioxidant activity [37,38]. The phenolic compounds content was measured by a colorimetric method using Folin–Ciocalteu reagent for each plant extract. Results were derived from a calibration curve (y = 9.7205x + 0.0325, R^2^ = 0.991) for gallic acid at different concentrations (10–250 µg/mL) and expressed as gallic acid equivalents (GAE) per gram of dry extract weight (Table 1). The total flavonoids content of the two extracts of *H. cinereum* (Table 1) are measured by using the aluminum chloride colorimetric method and the results were obtained from quercetin calibration curve (y = 0.0145x + 0.0099, R^2^ = 0.998).

The phenolic and flavonoid contents of the ethyl acetate extract showed a higher values (361.51 mg GAE/g; 148.23 ± 0.51 mg QE/g) than the butanol extract (145.88 mg GAE/g; 94.89 ± 0.29 mg QE/g). The total phenolic content (TPC) values observed in this study are higher than those reported by Benabdelaziz et al. for *H. sessiliflorum*, where the ethyl acetate and butanol extracts showed TPC values of 42.51 ± 1.01 mg GAE/g and 40.02 ± 2.81 mg GAE/g, respectively [14]. Moreover, the TPC values in this study exceed those found for the hydro-methanolic extract of *H. canum* (284.13 ± 0.30 mg GAE/g) [39]. Furthermore, total phenolic content values found in this study are higher than those of the ethyl acetate (46.70 ± 0.22 mg QE/g) and butanol (35.48 ± 0.36 mg QE/g) extracts of *H. sessiliflorum* and the hydro-methanolic extract of *H. canum* (13.13 ± 0.10 mg QE/g) [14,39].

### 2.3. Antioxidant Activities

Due to the complex activity of the phytoconstituents, the antioxidant activity of the extracts cannot be assessed by a single method, and it is recommended to use several methods, as each technique provides different and complementary information about the activity and the mechanism of action [40].

In this study, the antioxidant activity of *H. cinereum* extracts was evaluated using three different methods, as presented in Table 2. Across all methods (DPPH, FRAP, and ABTS), the ethyl acetate extract demonstrated stronger antioxidant capacity compared to the butanol extract. The antioxidant capacity shown by the DPPH method is notably higher than that reported by Benabdelaziz et al., for the ethyl acetate and butanol extracts of *H. sessiliflorum*, which had IC_50_ values of 23.75 ± 2.07 μg/mL and 94.03 ± 1.52 μg/mL, respectively [14]. Additionally, the IC_50_ values in this study were lower than those found for the hydro-methanolic extract of *H. canum* (0.19 mg/mL) [39], thus indicating the higher antioxidant activity.

**Figure 2:**
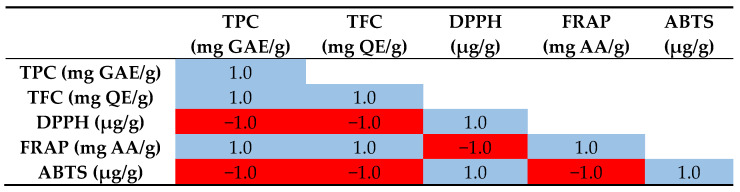
Pearson correlation matrix where red color indicates strong positive correlation and blue color indicates strong negative correlation.

The Pearson correlation results presented in Figure 2 demonstrate the exceptionally strong relationships between total phenolic content (TPC), total flavonoid content (TFC) and various measures of antioxidant activity (DPPH, FRAP and ABTS assays) in the analyzed samples. Analysis of the data shows that there is a perfect positive correlation between total phenolic and total flavonoid contents. This means that as the phenolic content in the samples increases, the flavonoid content increases proportionally. Phenolics and flavonoids, both subclasses of polyphenolic compounds, often co-occur and exhibit similar variations in plant materials. In addition, both TPC and TFC show a perfect positive correlation with the results of the Ferric Reducing Antioxidant Power (FRAP) assay. This suggests that higher levels of phenolics and flavonoids in samples are directly related to increased antioxidant power as measured by the FRAP assay. However, the total phenolic and flavonoid contents are perfectly negatively correlated with DPPH radical scavenging capacity and ABTS radical scavenging activity. If the DPPH and ABTS values correspond to IC₅₀ (the concentration needed to inhibit 50% of free radicals), a lower IC₅₀ value signifies greater antioxidant activity. Therefore, as the phenolic and flavonoid contents increase, the IC₅₀ values decrease, indicating increased antioxidant activity. This is consistent with the positive correlation between DPPH and ABTS. This means that samples with higher (or lower) DPPH radical scavenging capacity also have correspondingly higher (or lower) ABTS radical scavenging activity. Both assays measure the ability of antioxidants to scavenge free radicals, so they are expected to be highly correlated.

Finally, the FRAP values are perfectly negatively correlated with the DPPH and ABTS assay results. This indicates that samples with higher antioxidant power as measured by FRAP have lower IC₅₀ values in the DPPH and ABTS assays, which in turn indicates higher antioxidant activity. These findings from our study are supported by the previous studies that have shown that the capacity of the antioxidant is highly associated with the total flavonoid content and total phenolic compounds of the plant extract [41].

### 2.4. Antitumor Activity

#### 2.4.1. Cell Viability Assessment

**Figure 3:**
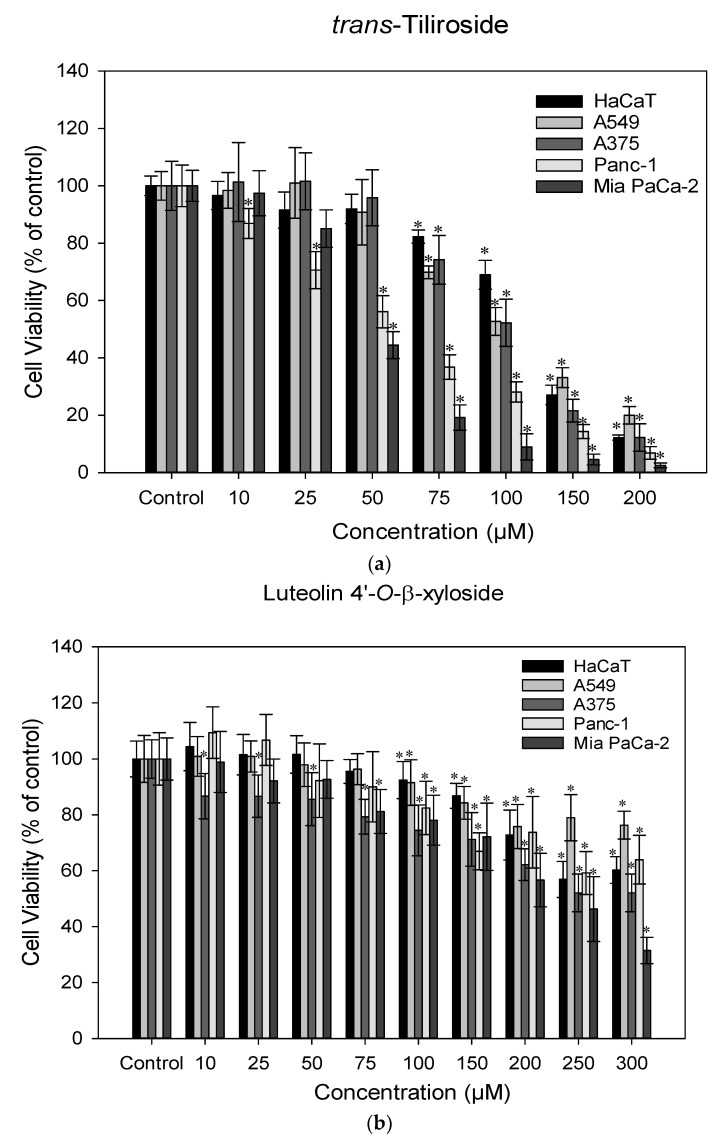
Effect of *trans*-tiliroside (**a**) and of luteolin 4′-*O*-β-xyloside (**b**) on the viability of HaCaT, A549, A375, Panc-1 and Mia PaCa-2 for 72 h exposure. Data shown are the mean values ± standard deviation of three independent assays with three technical replicates each. * Indicates statistical significance relative to control (*p* < 0.05).

The antitumoral activity of *trans*-tiliroside (**6**) and luteolin 4′-*O*-β-xyloside (**3**) was investigated against the lung cancer (A549), melanoma (A375), pancreatic cancer (Mia PaCa-2 and Panc-1) and immortalized human keratinocytes (HaCaT) cell lines by MTT assay. The MTT assay is a colorimetric assay based on the conversion of MTT into formazan crystals by living cells, which determines cellular metabolic activity, which is an indicator of cell viability [42]. As shown in Figure 3, all cell lines exhibited a decline in cell viability in a concentration-dependent manner. In the case of *trans*-tiliroside, Mia PaCa-2 and Panc-1 had a similar response, with Panc-1 having a decline of cell viability with the lowest tested concentration (10 μM), while Mia PaCa-2 was only affected by concentrations equal or above 50 μM. Notwithstanding, the determined IC_50_ was slightly lower to Mia PaCa-2 (46.2 ± 0.89 μM) than to Panc-1 (54.0 ± 1.95 μM). These results are consistent with a previous work that showed that *trans*-tiliroside can inhibit the growth of Panc-1 cells and determined a IC_50_ of 68.48 μM for 72 h of exposure [43]. Both the A549 and A375 cell lines showed to be more resistant to *trans*-tiliroside, having a significant decrease in cell viability when exposed to concentrations equal or superior to 75 μM and an IC_50_ of 108 ± 2.80 and 102 ± 2.56 μM, respectively. In the work of Lu et al. (2009), a decline of 80% in the viability of B16-F10 cells, another melanoma cell line, was observed after exposure of 168 μM of trans-tiliroside for 72 h [41]. Regarding HaCaT cells, the profile was very similar to A549 and A375 cells, obtaining an IC50 of 122 ± 1.80 μM [44].

Luteolin 4′-*O*-β-xyloside (**3**), on the other hand, had a more subtle effect on the four cell lines. The A375 cell line had a significant decrease in cell viability when exposed to concentrations up to 75 μM, contrary to the other three cell lines. Nevertheless, the literature reveals that luteolin aglycone has a strong antitumoral effect. In fact, studies suggest an IC_50_ between 7 and 17 μM for melanoma cell lines [45], and an IC_50_ values of 32.6 μM and 40 μM for the A549 cell line [46,47]. Pancreatic cancer cell lines also showed to be strongly affected by concentrations above 160 μM [48]. However, in our work, Mia PaCa-2 was the only cell line that showed a cell viability lower than 50% when exposed to the tested concentrations and the calculated IC_50_ was 229 ± 9.09 μM. Once again, HaCaT cells had a similar response to A375 cells at higher concentrations (250 and 300 μM) and were more resistant at lower concentrations (10–100 μM). Considering these results, both compounds seem to not be selective to cancer cells affecting normal cells equally.

#### 2.4.2. Cell Cycle Analysis

**Figure 4:**
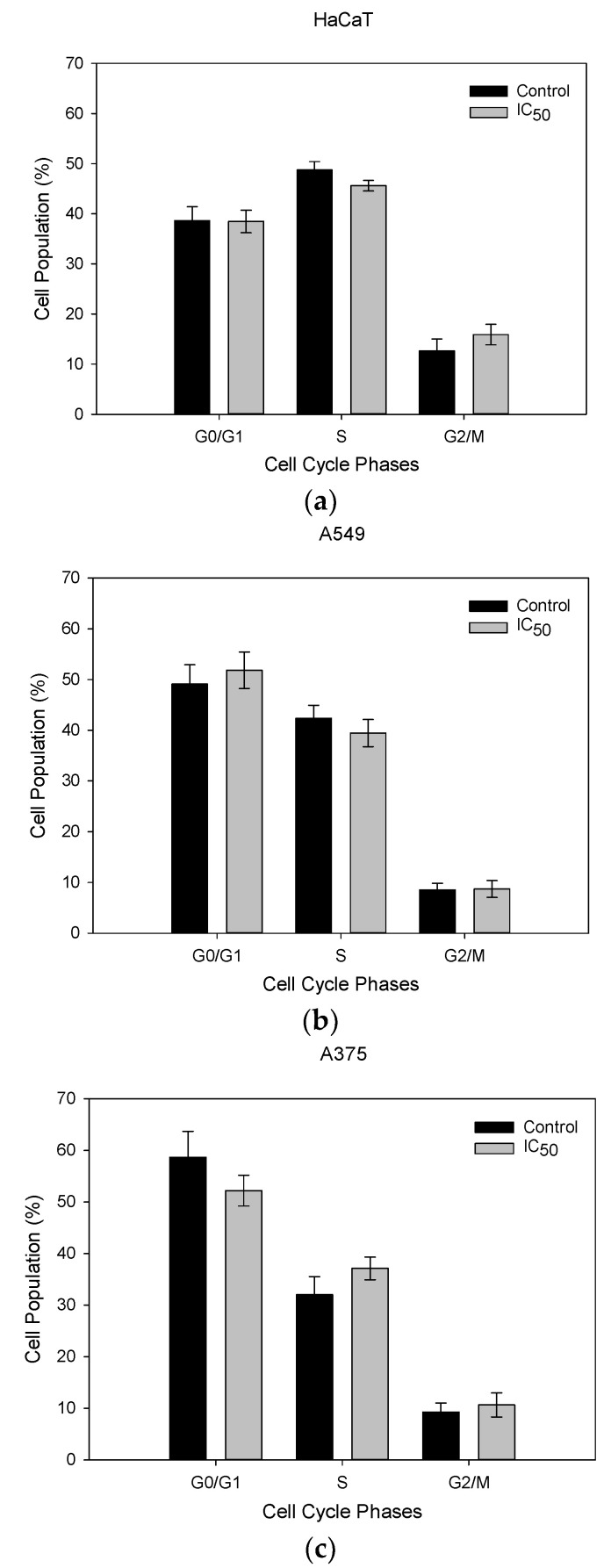

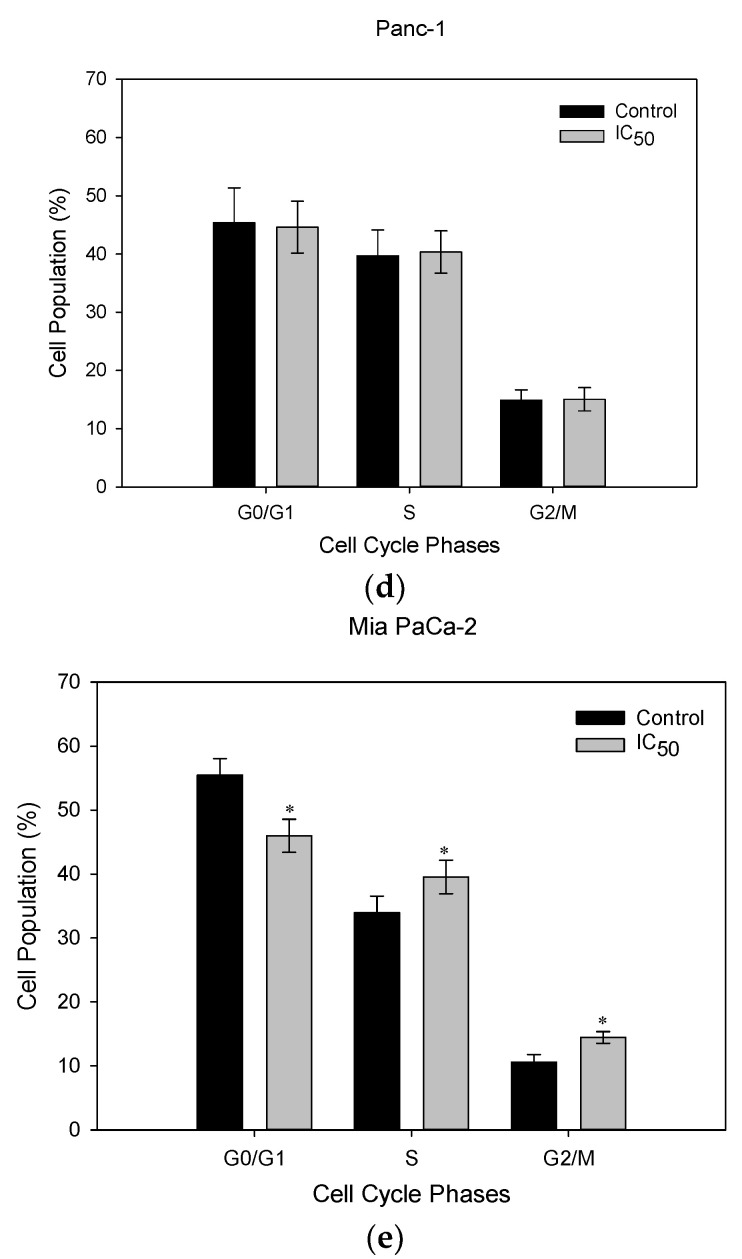
Cell cycle analysis of cells exposed to the respective IC_50_ of *trans*-tiliroside for 72 h. Data shown are the mean values ± standard deviation of two independent experiments, with two technical replicates each, and each replicate with at least 5000 events. * Indicates statistical significance relative to the control (*p* < 0.05).

The cell cycle is a fundamental process for cell proliferation [49]. To maintain genome integrity and prevent errors, it is tightly regulated by specific molecular checkpoints. These mechanisms can halt the cell cycle when DNA damage is detected, providing an opportunity for repair or triggering cell death, depending on the type and timing of the damage [50]. In cancer cells, however, these regulatory processes are disrupted, leading to uncontrolled proliferation. As a result, targeting the cell cycle has become a key strategy in cancer treatment, utilizing agents that interfere with these regulatory pathways. In our study, we investigated the impact of the IC_50_ of *trans*-tiliroside for 72 h treatment on cell cycle dynamics using flow cytometry. As seen in Figure 4, Mia PaCa-2 cells had a significant increase of 5.6% of cells at the S phase and of 3.9% G2/M phase when exposed to the treatment, compared to the control, potentially indicating an arrest at S phase and G2. HaCaT, A549, A375 and Panc-1 showed no alterations on the cell cycle dynamics.

## 3. Materials and Methods

### 3.1. Plant Material

The aerial parts of *Helianthemum cinereum* (Cav.) Pers were collected in the region of Oued El-ma, Merouana (Batna) during the flowering period in May 2018. The plant was identified by Pr. Bachir OUDJEHIH of the Department of Agronomy of the Institute of Veterinary and Agronomic Sciences from the University of Batna 1.

### 3.2. General Experimental Procedures

The NMR spectra were obtained on a Bruker 500 (Bruker, Billerica, MA, USA) (500 MHz for ^1^H and 125 MHz for ^13^C) and a Bruker 300 (300 MHz for ^1^H and 75 MHz for ^13^C) spectrometers (Bruker, Billerica, MA, USA). All 1D and 2D NMR experiments were performed using the standard Bruker library of microprograms. The spectra were recorded in deuterated methanol. Chemical shifts (δ) are in part per million (ppm) relative to Tetramethylsilane (TMS, Sigma-Aldrich, St. Louis, MO, USA) and Coupling constant (*J*) are in Hz. referenced solvent peaks of CD_3_OD (Sigma-Aldrich, St. Louis, MO, USA): ^1^H δ 3.31, ^13^C δ 49.1.

Electrospray ionization mass spectra were acquired with a Micromass Q-Tof 2 (Micromass, Manchester, UK), operating in positive ion mode, equipped with a Z-spray source, an electrospray probe, and a syringe pump. The source and desolvation temperatures were 80 and 150 °C, respectively. The capillary voltage was 3000 V. The spectra were acquired at a nominal resolution of 9000 and at cone voltages of 30 V. Nebulization and collision gases were N_2_ and Ar, respectively. Compound solution in methanol were introduced at a 10 μL min^−1^ flow rate.

### 3.3. Extraction and Isolation

One kilogram of the dried part of aerial parts of H. cinereum was extracted three times (3 × 10 L) with a mixture of 70% EtOH (Sigma-Aldrich, Steinheim, Germany) in water at room temperature (22–25 °C). After evaporation of the solvent, the resulting aqueous solution (300 mL) was successively partitioned three times with 200 mL of petroleum ether (PE) (Sigma-Aldrich, Steinheim, Germany), 200 mL of ethyl acetate (AcOEt) (Sigma-Aldrich, Steinheim, Germany) and 200 mL of butanol (BuOH) (Sigma-Aldrich, Steinheim, Germany), to obtain, after removal of the solvents in vacuum, three organic extracts: PE (5.0 g), EtOAc (10.0 g), and BuOH (40.0 g). The AcOEt extract (10.0 g) was first chromatographed on a silica gel column eluting with a gradient solvent of CHCl_3_/MeOH (Sigma-Aldrich, Steinheim, Germany), starting with 100 mL of CHCl_3_ and progressively increasing the proportion of 10% MeOH in CHCl_3_ as the eluent, adding 100 mL increments until the mixture reaches a composition of 60% MeOH in CHCl_3_, to give nine fractions (F1 to F9).

F6 (810 mg) was applied to a silica gel column chromatography, eluted with CHCl_3_/MeOH to give sixteen subfractions (F6-1 to F6-16). Elution was carried out using 100 mL of CHCl_3_, followed by successive 100 mL increments of 5% MeOH in CHCl3, gradually increasing up to 50% MeOH. Subfraction F6-8 (92 mg) was then chromatographed over Sephadex LH-20 column (Merck, Darmstadt, Germany), and eluted with isocratic elution system CHCl_3_/MeOH (1/1, *v/v*, 150 mL) to yield five further subfractions (F6-8-1 to F6-8-5). Subfraction F6-8-3 (40 mg) was purified by using RP-18 SPE (Waters, Milford, MA, USA) cartridge eluted with increasing proportions of a gradient MeOH in H_2_O (0, 10, 30, 50 and 100%), to afford compounds (**3**) (4 mg), (**6**) (15 mg), (**7**) (4 mg), and (**10**) (5 mg). Fractions eluted with MeOH/H_2_O (80:20) gave compound (**3**) while compounds (**6**), (**7**), and (**10**) were obtained from fractions eluted with 85%, 5% and 10% MeOH in H_2_O, respectively.

The final subfraction, F6-8-5, underwent further purification using an RP-18 SPE cartridge and was eluted with a MeOH/H_2_O gradient (from 0% to 90% MeOH). This process yielded compounds (**1**) (1.4 mg), (**2**) (8 mg), (**5**) (1.4 mg), (**8**) (21 mg), and (**9**) (1.6 mg). Compounds (**1**) and (**2**) were obtained from the 95% MeOH fractions, while compounds (**5**), (**8**) and (**9**) were obtained from the 80%, 5% and 10% MeOH fractions, respectively. F7 (2 g) was subjected to SiO_2_ gel column chromatography and sequentially eluted with 10%, 20%, 30%, 40%, 50%, 70%, and 100% MeOH in CHCl_3_, resulting in ten subfractions designated as F7-1 to F7-10.

Subfraction F7-9 (400 mg) was continually purified on Sephadex LH-20 column with isocratic system (CHCl_3_ /MeOH: 1/1, *v/v*, 200 mL) to obtain eight subfractions (F7-9-1 to F7-9-8). The third fraction in this series (F7-9-3) was further purified using an RP-18 SPE cartridge, eluted with H_2_O/MeOH at concentrations of 10%, 30%, 50%, and 100%. Fractions eluted with 80% MeOH in H_2_O yielded compound (**4**) (2 mg).

Following the same purification method, the polar butanol extract of H. cinereum was initially processed through RP-18 column chromatography using pure MeOH to eliminate free sugars. The methanolic solution was concentrated and a mass of nine grams was purified by using vacuum liquid chromatography VLC (RP-18, 100 g). Elution began with 100 mL of H₂O, followed by 100 mL increments of H₂O with progressively increasing MeOH concentrations (10% increments) until reaching 50% MeOH in H₂O. The fraction eluted with water (4 g) was subjected to a silica gel column chromatography by using a gradient of CH_2_Cl_2_ (Sigma-Aldrich, Steinheim, Germany) in MeOH to afford fifteen fractions (F1-F15). Subfraction F9 (90 mg) was further separated on RP-18 SPE cartridge and eluted with a gradient of MeOH in H_2_O to afford six subfractions (F2-1 to F2-6). Subfraction F2-3 (25 mg) was purified on Sephadex LH-20 column using an isocratic system MeOH–CHCl_3_ (1/1, *v/v*, 200 mL) to give compound (**11**) (3 mg). However, the fifth subfraction, F2-5 (8.1 mg), was purified using an RP-18 SPE cartridge and eluted with a gradient H₂O/MeOH at concentrations of 10%, 30%, 50%, and 100%, Fractions eluted with 5% MeOH yielded pure compound (**12**) (0.5 mg).

**Compound 3:** Luteolin 4′-*O*-β-xyloside, yellow amorphous solid: ^1^H-NMR (500 MHz, CD_3_OD) δH (ppm): aglycone 7.47 (^1^H, dd, *J* = 8.0 and 2.2 Hz, H6′), 7.45 (^1^H, s, H-2′), 7.25 (^1^H, d, *J* = 9.1 Hz, H-5′), 6.62 (^1^H, s, H-3), 6.45 (^1^H, d, *J* = 2.0 Hz, H-8), 6.21 (^1^H, d, *J* = 2.0 Hz, H-6), xylose: 4.94 (^1^H, overlap, H-1″), 4.0 (^1^H, dd, *J* = 11.5 and 5.1 Hz, H-5a′′), 3.63 (^1^H, m, H-4″), 3.57 (^1^H, dd, *J* = 7.5 Hz, H-2″), 3.50 (^1^H, t, *J* = 8.3 Hz, H-3″), 3.43 (^1^H, d, *J* = 11.3 Hz, H-5b″); ^13^C-NMR (125 MHz, CD_3_OD) δC (ppm): aglycone 183.7 (C, C-4), 165.0 (C, C-2), 158.8 (C, C-5), 164.1 (C, C-7), 158.7 (C, C-9), 149.9 (C, C-4′), 148.1 (C, C-3′), 105.1(C, C-3), 126.7 (C, C1′), 119.9 (CH, C-6′), 116.5 (CH, C-5′), 113.5 (CH, C-2′), 105.3 (C, C-10), 93.8 (CH, C-8), xylose: 102.3 (CH, C-1″), 75.8 (CH, C-3″), 73.2 (CH, C-2″), 69.5 (CH, C-4″), 65.5 (CH, C-5″).

**Compound 4:** Luteolin 4′-*O*-β-glucoside, yellow amorphous solid: ^1^H-NMR (500 MHz, CD*_3_*OD) δH (ppm) aglycone: 7.49 (^1^H, dd, *J* = 8.6 and 2.1 Hz, H-6′), 7.47(^1^H, d, *J* = 2.1 Hz, H-2′), 7.34 (^1^H, d, *J* = 8.5 Hz, H-5′), 6.64 (^1^H, s, H-3), 6.48 (^1^H, d, *J* = 2.1 Hz, H-8), 6.23 (^1^H, d, *J* = 2.1 Hz, H-6), glucose: 4.96 (^1^H, d, *J* = 8.0 Hz, H-1′′), 3.94 (^1^H, dd, *J* = 12.2 and 2.0 Hz, H-6a″), 3.75 (^1^H, dd, *J* = 12.2 and 5.6 Hz, H-6b″), 3.56 (^1^H, t, *J* = 7.2 Hz, H-2″), 3.52 (^1^H, m, H-5″), 3.50 (^1^H, m, H-3″), 3.45 (^1^H, t, *J* = 9.4 Hz, H-4″); ^13^C-NMR (125 MHz, CD_3_OD) δC (ppm) aglycone: 182.5 (C, C-4), 164.8 (C, C-2), 164.2(C, C-7), 161.8(C, C-5), 158.1(C, C-9), 148.6 (C, C-4′), 147.2 (C, C-3′), 125.8 (C, C-1′), 119.5 (CH, C-6′), 117.5 (CH, C-5′), 114.5 (CH, C-2′), 104.1 (C, C-10), 104.8 (CH, C-3), 99.8 (CH, C-6), 94.8 (CH, C-8), glucose: 102.7 (CH, C-1″), 76.1 (CH, C-5″), 77.1 (CH, C-3″), 73.3 (CH, C-2″), 69.8 (CH, C-4″), 62.2 (CH, C-6′′).

**Compound 5:** Quercetin 4′-*O*-β-xyloside, yellow crystal: ^1^H-NMR (500 MHz, CD_3_OD) δH (ppm) aglycon: δH 7.80 (^1^H, d, *J*=2.0 Hz, H-2′), 7.73 (^1^H, dd, *J* = 8.8, 2.0 Hz, H-6′), 7.23 (^1^H, d, *J*=8.8 Hz, H-5′), 6.42 (^1^H, d, *J* = 2.0 Hz, H-8), 6.21 (^1^H, d, *J* = 2.0 Hz, H-6), xylose: 4.91 (^1^H, d, *J* = 7.8 Hz, H-1″), 3.99 (^1^H, dd, *J* = 11.3 and 5.3 Hz, H-5″a), 3.63 (^1^H, m, H-4″), 3.55 (^1^H, m, H-2″), 3.48 (^1^H, m, H-3″), 3.42 (^1^H, m, H-5b″); ^13^C NMR (125 MHz, CD_3_OD) δC (ppm) aglycone: 176.2 (C, C-4), 164.7 (C, C-7), 161.9 (C, C-9), 158.8 (C, C-5), 146.5 (C, C-4′), 146.4 (C, C-3′), N.D (C, C-3), 126.4 (C, C-1′), 119.8 (CH, C-6′), 116.0 (CH, C-5′), 115.5 (CH, C-2′), 103.1 (C, C-10), 98.0 (CH, C-6), 93.1 (CH, C-8), xylose: 102.5 (CH, C-1″), 75.9 (CH, C-3″), 73.2 (CH, C-2″), 69.6 (CH, C-4″), 65.6 (CH_2_, C-5″).

**Compound 11:** Shikimic acid 3-*O*-gallate, white powder: ^1^H NMR (500 MHz, CD_3_OD): δH 6.84 (^1^H, s, H-2), 5.32 (^1^H, dt, *J* = 5.2 Hz, H-5), 4.51 (^1^H, t, *J* = 3.8 Hz, H-3), 4.04 (^1^H, dd, *J* = 3.5, H-4), 2.88 (^1^H, dd, *J* = 5.2 and 18.5 Hz, H-6a), 2.40 (^1^H, dd, *J* = 5.2 and 18.5 Hz, H-6b); galloyl-H: 7.12 (2H, s, H-2′/H-6′), ^13^C NMR (125 MHz, CD_3_OD): 171.4 (C, C-7), 137.5 (CH, C-2), 131.2 (C, C-1), 71.6 (CH, C-5), 69.4 (CH, C-4), 66.9 (CH, C-3), 28.7 (CH_2_, C-6), galloyl: 168.1 (-OCO-), 121.2 (C, C-1′), 110.4 (2CH, C-2′/C-6′), 145.9 (2CH, C-3′/C-5′), 139.5 (CH, C-4′).

**Compound 12:** 3,3′,4′-tri-*O*-methylellagic acid sulphate, amorphous: ^1^H NMR (500 MHz, CD_3_OD): δ 8.36 (^1^H, s, H-5), 7.78 (^1^H, s, H-5′), 4.30 (3H, s, OCH_3_-3), 4.17 (3H, s, OCH_3_-3′), 4.08 (3H, s, OCH_3_-4′), ^13^C NMR (125 MHz, CD_3_OD): 159,1 (C-9), 155.2 (C, C-4′), 145.0 (C, C-3), 141.8 (C, C-3′), 119.3 (CH, C-5), 115.8 (C, C-1), 112.9 (C, C-1′), 108.6 (CH, C-5′), 62.2 (OCH_3_, C-3), 61.8 (OCH_3_, C-3′), 56.9 (OCH_3_, C-4′).

### 3.4. Determination of Total Phenolic Compounds (TPC)

The total phenolic compounds (TPC) amount in *H. cinereum* extract was determined by the Folin–Ciocalteu method as described by [51]. Firstly, extract was diluted (50 times in Ethanol) due to its high concentration. Then, aliquot of 0.5 mL was mixed with 2.5 mL of 10% (*v*/*v*) Folin–Ciocalteu reagent (Sigma-Aldrich, Steinheim, Germany). After 8 min in the dark, 2 mL of sodium carbonate Na_2_CO_3_ (7.5%, *w*/*v*) (Sigma-Aldrich, Steinheim, Germany) was added, and the reaction was carried out in the dark for 1 h. Then, the absorbance at 765 nm was measured using a UV–Vis spectrophotometer (UV 1700, Shimadzu, Japan). Gallic acid standard curve was built by the preparation of five concentrations of gallic acid (10, 50, 75, 100 and 250 µg/g), starting from a stock solution of 5 mg/g. TPC concentration was obtained from the calibration curve and expressed as mg gallic acid equivalent per gram of dried sample weight (mg GAE/g dry weight).

### 3.5. Determination of Total Flavonoids Compounds (TFC)

Total flavonoids content of *H. cinereum* extract was determined by the aluminum chloride colorimetric method as described by [52] with some modifications, 0.5 mL of extract in methanol was mixed with 0.1 mL of aluminum chloride solution (10%), 0.1 mL of potassium acetate (1 M) and 4.3 mL of distilled water. The mixture was incubated at room temperature for 30 min. Then, the absorbance was measured at 415 nm using a UV–Vis spectrophotometer (UV 1700, Shimadzu, Japan), and the flavonoid content was calculated based on a quercetin calibration curve, obtained by preparation of a serial concentrations (5 to 60 µg/g). All experiments were performed in triplicate and averaged; the results are expressed in mg of quercetin equivalent per gram of dry extract (mg QE/g).

### 3.6. Antioxidant Activity

#### 3.6.1. DPPH Radical Scavenging Capacity

The free radical scavenging activity of *H. cinereum* extracts was investigated by the 2,2-Di(4-tert-octylphenyl)-1-picrylhydrazyl (DPPH) method as described by [53] with some modifications. Different concentrations of *H. cinereum* extract were prepared in methanol and aliquots of 100 μL were added to 3.5 mL of DPPH (Sigma-Aldrich, Steinheim, Germany) solution (30 μg/g in methanol). A blank consisting of DPPH solution without extract was prepared. All samples were kept in darkness for 15 min. Then, the absorbance was measured against a blank (Methanol) at 515 nm in a UV–Vis spectrophotometer (UV 1700, Shimadzu, Japan). The DPPH solution was daily prepared and the concentration was checked using a calibration curve obtained by preparation of serial concentrations (4, 8, 16, 32 and 64 μg/g).

The antioxidant capacity of the samples was expressed as DPPH percentage of inhibition (I%), calculated by the Equation (1):

(1) $$I\%=\frac{A_{0}-A}{A_{0}}\times 100$$

where A_0_ and A represent the absorbance values of blank (DPPH with methanol) and the plant extract sample (DPPH with extract), respectively. The values of the inhibition percentage after 30 min were plotted versus the extract concentration to obtain the antioxidant capacity curve, and a linear regression was designed to obtain the IC_50_ value (concentration giving a reduction of 50% of DPPH concentration). The obtained IC_50_ value is inversely proportional to the antioxidant activity.

#### 3.6.2. Ferric Reducing Antioxidant Power (FRAP) Assay

The iron reducing capacity of the *H. cinereum* extracts was determined by the method described by [54], with some modifications. The 1 mL aliquots consisting of various dilutions of extract in methanol were added to 2.5 mL of phosphate buffer (0.2 M, pH 6.6), and 2.5 mL of potassium ferricyanide (1%) (Sigma-Aldrich, St. Louis, MO, USA). Then, the mixture was incubated at 50 °C for 20 min and 2.5 mL of trichloroacetic acid (10%) was added to the sample and centrifuged at 3000 rpm for 10 min. After that, a volume of 2.5 mL of the mixture supernatant was added to 2.5 mL of distilled water and 0.5 mL ferric chloride (0.1%) and vigorously mixed. Then, the absorbance of the sample is measured using a UV–Vis spectrophotometer (UV 1700, Shimadzu, Japan) at 700 nm against a blank similarly prepared by replacing the plant extract with methanol.

Ascorbic acid (Merck, Darmstadt, Germany) calibration curve was built by preparation of a serial concentrations (0.001, 0.025, 0.05, 0.1 and 0.2 mg/g), the results are expressed in milligrams equivalent of ascorbic acid per gram of extract.

#### 3.6.3. ABTS Assay

The ABTS radical scavenging activity of *H. cinereum* extracts was assessed according to the method described by [55]. Firstly, 1 mL of ABTS (Sigma-Aldrich, Steinheim, Germany) stock solution (7 mM) was mixed with 88 μL of potassium persulfate solution (140 mM) and kept overnight protected from light and at room temperature. Next, the mixture was diluted with potassium buffer saline (0.2 M, pH 7.4) to obtain an ABTS solution with an absorbance of 0.70 ± 0.02 at 734 nm. Next, a volume of 3 mL of this ABTS solution was mixed with 1 mL of the sample at different concentrations (25, 50, 75, 100 and 250 µg/mL). The reaction took place in the dark for 6 min, and the absorbance at 734 nm was measured. Samples containing the same volume of methanol and the same dilution of ascorbic acid served as white and positive controls, respectively. ABTS free radical scavenging capacity was determined using the Equation (2):

(2) $$ABTS\;radical\;scavenging\;activity\;\left(\%\right)=1-\frac{A_{1}-A_{2}}{A_{0}}\times 100$$

where A_1_ is ABTS radical and sample mixture absorbance; A_2_ is the solvent and sample mixture absorbance and A_0_ is the absorbance of the solvent and ABTS radical mixture.

### 3.7. Antitumoral Activity

#### 3.7.1. Cell Culture

The immortalized human keratinocytes HaCaT cells were obtained from Cell Lines Services (Eppelheim, Germany). The A549 cell lines were acquired from the European Collection of Authenticated Cell Cultures (ECACC) and supplied by Sigma-Aldrich (St. Louis, MO, USA), the A375 cell lines were acquired from ATCC and supplied by LGC Standards (Barcelona, Spain), and Mia PaCa-2 and Panc-1 cell lines were kindly provided by Dr. Sónia Melo (i3s, Porto, Portugal). All cell lines were maintained in High-Glucose Dulbecco’s Modified Eagle’s Medium (DMEM, PAN-Biotech, Aidenbach, Germany), supplemented with 10% (*v/v*) fetal bovine serum (FBS, PAN-Biotech, Aidenbach, Germany), 2 mM L-glutamine, and 1% pen/strep (100 U/mL penicillin, 100 ug/mL streptomycin (Grisp, Porto, Portugal). Cells were cultured at 5% CO_2_ and at 37 °C.

#### 3.7.2. Cell Viability Assessment

A stock solution was made by dissolving *trans*-tiliroside or luteolin 4′-*O*-β-xyloside in dimethyl sulfoxide (DMSO, Sigma-Aldrich, St. Louis, MO, USA). All cell lines were seeded in 96-well plates at a density of 12,500 cells/mL and incubated for 24 h for adhesion. Next, cells were exposed to a range of concentrations of trans-tiliroside (10, 25, 50, 75, 100, 150, 200 μM) or of luteolin 4′-*O*-β-xyloside (10, 25, 50, 75, 100, 150, 200, 250, 300 μM) for 72 h. After the exposure, cell viability was assessed by 3-(4,5-dimethyl-2-thiazolyl)-2,5-diphenyl-2H-tetrazolium bromide (MTT, Sigma-Aldrich, St. Louis, MO, USA) assay. Briefly, the exposure medium was replaced by fresh medium and 50 μL of MTT (1.0 mg/mL dissolved in phosphate-buffered saline, PBS, PAN-Biotech, Aidenbach, Germany) and plates were incubated for 3 h. Then, the medium was removed and 150 μL of DMSO was added and plates were shaken in the dark for 1 h. The absorbance was read in a microplate reader BioTek Synergy HT plate reader at 570 nm. Cells with only medium without compounds were used as control. Three independent assays were completed with three replicates each. Cell viability was further calculated using the Equation (3):

(3) $$Cell\;Viability\;\left(\%\;of\;control\right)=\frac{Sample\;Absorbance-Blank\;Absorbance}{Control\;Absorbance-Blank\;Absorbance}\times 100$$

#### 3.7.3. Cell Cycle Assessment

Cells were seeded in 12-well plates as described before. After adhesion, the medium was replaced by *trans*-tiliroside at the equivalent concentrations of IC_50_ of each cell line (122, 108, 102, 54 and 46.2 μM for HaCaT, A549, A375, Panc-1 and Mia PaCa-2, respectively) for 72 h. Then, cells were collected, washed with PBS, fixed with 85% ethanol and stored at −20 °C until analysis. For analysis, cells were washed, resuspended in PBS and filtered to Eppendorf tubes. Samples were incubated with 50 μL of RNAse (Sigma-Aldrich, St. Louis, MO, USA) for 10 min and next with 50 μL of propidium iodide (PI, ≥94%; Sigma-Aldrich, St. Louis, MO, USA) for 20 min. Cells were analyzed with an Attune^®^ Acoustic Focusing Cytometer (Applied Biosystems, Termo Fischer Scientific, Agawam, MA, USA) and the percentages of cells at each phase of the cell cycle were determined using the FlowJo software V10.7.1 (FlowJo LLC, Ashland, OR, USA). Two independent assays with two replicates each were performed, with at least 5000 events.

### 3.8. Statistical Analysis

For the TPC and TFC determinations and antioxidant activity, each sample was analyzed three times. The data presented are the means of the results obtained. The errors are expressed as standard deviations. Student’s *t*-test was used to determine significant differences (*p* ≤ 0.05) between the samples analyzed. The null hypothesis—that the samples are identical—was first considered. The experimental t values were compared with the theoretical t values. If the two values were equal or the experimental value was lower than the theoretical value, the null hypothesis was accepted. Conversely, if the experimental value was higher than the theoretical value, the null hypothesis was rejected, leading to the conclusion that the samples were significantly different.

In addition, the Pearson correlation matrix was performed using the Data Analysis Tools of Microsoft Excel version 2408. The Pearson correlation matrix is a table showing the Pearson correlation coefficients between pairs of variables. The Pearson correlation coefficient, denoted as r, measures the linear relationship between two continuous variables, indicating how strongly they are related and in which direction (positive or negative). Values of r range from −1 to 1.

Perfect positive correlation, meaning that as one variable increases, the other also increases in a perfectly linear manner, is obtained when r = 1. A perfect negative correlation, which means that as one variable increases, the other decreases in a perfectly linear manner, is obtained when r = −1.

Finally, when r = 0, there is no linear correlation, indicating that there is no linear relationship between the variables.

For the antitumor activity, the results are represented as the mean ± standard deviation. SigmaPlot version 14.0 (Systat Software, San Jose, CA, USA) for Windows was used to for statistical analysis. Data were analyzed by one-way ANOVA (*p* < 0.05) followed by Dunnett’s test. The differences were considered statistically significant for *p* < 0.05.

## 4. Conclusions

The present study, which is focused on the phytochemical study and the biological activities of the Algerian species *Helianthemum cinerum*, allowed us to isolate twelve compounds from the ethyl acetate (EtOAc) and butanol (BuOH) extracts. All the compounds were identified by spectral analysis, mainly through NMR experiments (¹H, ¹³C, COSY, HSQC, and HMBC) and mass spectrometry, as well as by comparing their spectroscopic data with those reported in the literature. To the best of our knowledge, this is the first report of six compounds being isolated from the Cistaceae family—luteolin 4′-*O*-β-xyloside (**3**), luteolin 4′-*O*-β-glucoside (**4**), quercetin 4′-*O*-β-xyloside (**5**), ethyl gallate (phyllemblin) (**10**), shikimic acid 3-*O*-gallate (**11**), and 3,3′,4′-tri-*O*-methyl-ellagic acid 4-sulfate (**12**). Further, the isolated compounds *trans*-tiliroside and luteolin 4′-*O*-β-xyloside were assessed for their antitumor activity against the lung cancer (A549), melanoma (A375), pancreatic cancer (Mia PaCa-2 and Panc-1), and immortalized human keratinocytes (HaCaT) cell lines by MTT assay, and cell cycle analysis, where we noticed an important activity and cell lines showed a concentration-dependent decrease in cell viability.

In addition to determining total flavonoid and phenolic contents (TFC and TPC), the extracts were evaluated for their antioxidant capacity using in vitro free radical scavenging assays (DPPH, ABTS) and the FRAP assay. The results of antioxidant capacity revealed that the ethyl acetate (EtOAc) extract exhibited stronger activity than the butanol (BuOH) extract; however, considering the available data, the potential of both extracts remains higher compared to extracts from other species of the same genera. Correspondingly, a positive correlation between total phenolic and flavonoid contents and the antioxidant activity of the investigated extracts indicated that these phytoconstituents are the major contributors to the antioxidant capacities of this plant. These findings suggest that *H. cinereum* could be a promising source of natural antioxidants. Further complementary experiments focusing on other biological activities should be conducted to evaluate the potential of various compounds isolated from the plant. Additionally, optimizing the extraction procedures for the most significant phytoconstituents is crucial for potential future applications.

## Figures and Tables

**Table 1:** Total phenolic and flavonoid contents of *H. cinereum* extracts.

Extracts	TPC (mg GAE/g) *	TFC (mg QE/g) **
*H. cinereum* EtOAc	361.51 ± 0.84 ^a^	148.23 ± 0.51 ^a^
*H. cinereum* BuOH	145.88 ± 0.63 ^b^	94.89 ± 0.29 ^b^

* GAE—gallic acid equivalents; ** QE—quercetin equivalents. a, b—different letters indicate significantly (*p* ≤ 0.05) different results according to *t*-tests.

**Table 2:** Antioxidant activity of *H. cinereum* extracts.

Samples	DPPH (IC_50_ µg/g) **	FRAP (mg AA/g) *	ABTS (IC_50_ µg/g) **
*H. cinereum* EtOAc	17.23 ± 0.36 ^a^	221.16 ± 1.03 ^a^	85.16 ± 1.03 ^a^
*H. cinereum* BuOH	24.39 ± 0.21 ^b^	44.69 ± 0.64 ^b^	121.16 ± 1.03 ^b^
Trolox	11.97 ± 0.41	-	23.16 ± 0.54
Ascorbic acid	3.36 ± 0.13	-	-

* AA—ascorbic acid; ** IC—inhibition concentration. a, b—different letters indicate significantly (*p* ≤ 0.05) different results according to *t*-tests.

## Data Availability

Supplementary data related to this study are available and can be downloaded at the journal website.

## Supplementary Materials

The following supporting information can be downloaded at: https://www.mdpi.com/article/10.3390/molecules29245935/s1, NMR spectrum of the compounds (**1**–**12**); Mass spectrum of the compounds (**3**, **4**, **5**, **11** and **12**).
