# Supplementary material for: Phytochemical Study and In Vitro Antioxidant Activity of Helianthemum cinereum Along with Antitumor Activity of the Isolated trans-Tiliroside and Luteolin 4′-O-β-Xyloside
Source: Molecules. 2024 Dec 16;29(24):5935. doi: 10.3390/molecules29245935 (PMC11678426; doi:10.3390/molecules29245935)
Supplement: Supplementary file 1 [file molecules-29-05935-s001.zip › molecules-3344198-supplementary.pdf]

Supporting Information (2)

Phytochemical Study and In Vitro Antioxidant Activity of Helianthemum cinereum Along with Antitumor Activity of the Isolated Trans-Tiliroside and Luteolin 4′-O β-Xyloside

Anis Bertella[1], Abla Smadi[2], Hakim Benhabrou[2], Diana Salvador[3], Magdalena Wrona[4], Helena Oliveira[3], Abouamama Sidaoui[5], Georgiana Gavril-Luminita[6], Diana Pinto[7], Ewa Olewnik-Kruszkowska[8], Cristina Nerín[4], Artur Silva[7] and Fatma Bitam [2,9].

[1] *Department of Molecular and Cellular Biology, Faculty of Life and Nature Sciences, Abbes Laghrour University Khenchela, BP 1252 Road of Batna, Khenchela 40004, Algeria; anis.bertella@univ-khenchela.dz*

[2] *Department of Chemistry, Faculty of Matter Sciences, Laboratory of Chemistry and Environmental Chemistry (LCCE), University of Batna 1, 05000, Algeria, abla.smadi@univ-batna.dz ; hakim.benhabrou@univ-batna.dz*

[3] *Department of Biology & CESAM, University of Aveiro, Aveiro, Portugal; holiveira@ua.pt*

[4] *Departmento de Química Analítica, Instituto de Investigación en Ingeniería de Aragón (I3A), Escuela de Ingeniería y Arquitectura (EINA), Universidad de Zaragoza, María de Luna 3 (Edificio Torres Quevedo), 50018 Zaragoza, Spain; magdalenka.wrona@gmail.com (M.W.); cnerin@unizar.es (C.N.)*

[5] *Faculty of Sciences and Technology, Department of Biology, Amine Elokkal El Hadj Moussa Egakhamouk University of Tamanghasset, Tamanghasset 11000, Algeria; a.sidaoui@univ-tam.dz*

[6] *Department of Bioinformatics, National Institute of Research and Development for Biological Sciences, 296 Splaiul Independentei, Sector 6, 060031 Bucharest, Romania; georgi.gavril@yahoo.com*

[7] *LAQV/REQUIMTE, Department of Chemistry, Campus Universitário de Santiago, University of Aveiro, 3810-193 Aveiro, Portugal; diana@ua.pt (D.C.G.A.P.); artur.silva@ua.pt (A.M.S.S.)*

[8] *Physical Chemistry and Physicochemistry of Polymers, Faculty of Chemistry, Nicolaus Copernicus University in Torún, Gagarin 7 Street, 87-100 Torún, Poland; olewnik@umk.pl*

[9] *Faculte De Medecine, Departement De Pharmacie, Universite De Batna 2, 05000, Algeria ; f.bitam@univ-batna2.dz*

corresponding author

f.bitam@univ-batna2.dz

S1. 1H NMR spectrum of compound (3) (300 MHz, CD3OD)

S2. 13C NMR spectrum of compound (3) (75 MHz, CD3OD)

S3. HSQC spectrum of compound (3) (500 MHz, CD3OD)

S4. HMBC spectrum of compound (3) (500 MHz, CD3OD)

S5. 1H NMR spectrum of compound (4) (500 MHz, CD3OD)

S6. 13C NMR spectrum of compound (4) (125 MHz, CD3OD)

S7. HSQC spectrum of compound (4) (500 MHz, CD3OD)

S8. HMBC spectrum of compound (4) (500 MHz, CD3OD)

S9. 1H NMR spectrum of compound (5) (300 MHz, CD3OD)

S10. 13C NMR spectrum of compound (5) (75 MHz, CD3OD)

S11. HSQC spectrum of compound (5) (500 MHz, CD3OD)

S12. HMBC spectrum of compound (5) (500 MHz, CD3OD)

S13. 1H NMR spectrum of compound (6) (600 MHz, CD3OD)

S14. 1H NMR spectrum of compound (11) (500 MHz, CD3OD)

S15. COSY spectrum of compound (11) (500 MHz, CD3OD)

S16. 13C NMR spectrum of compound (11) (125 MHz, CD3OD)

S17. HSQC spectrum of compound (11) (500 MHz, CD3OD)

S18. HMBC spectrum of compound (11) (500 MHz, CD3OD)

S19. 1H NMR spectrum of compound (12) (500 MHz, CD3OD)

S20. HSQC spectrum of compound (12) (500 MHz, CD3OD)

S21. HMBC spectrum of compound (12) (500 MHz, CD3OD)

Compound (3): Luteolin 4'-*O*-β-xyloside

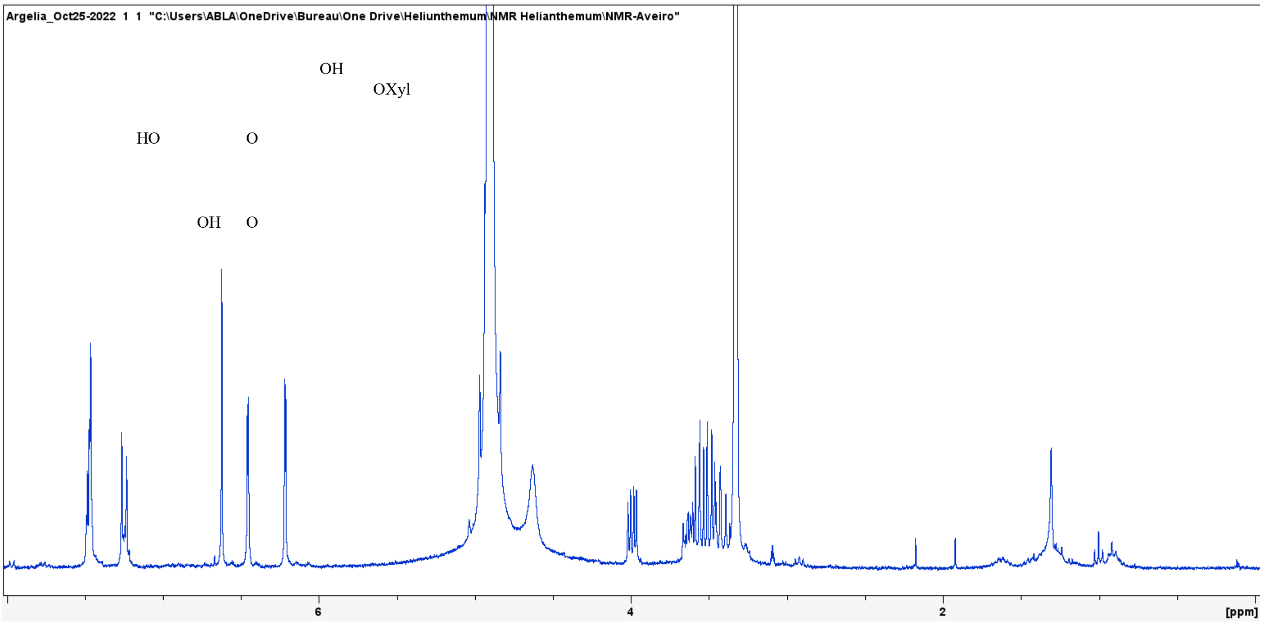

S1. 1H NMR spectrum of compound 3 (500 MHz, CD3OD)

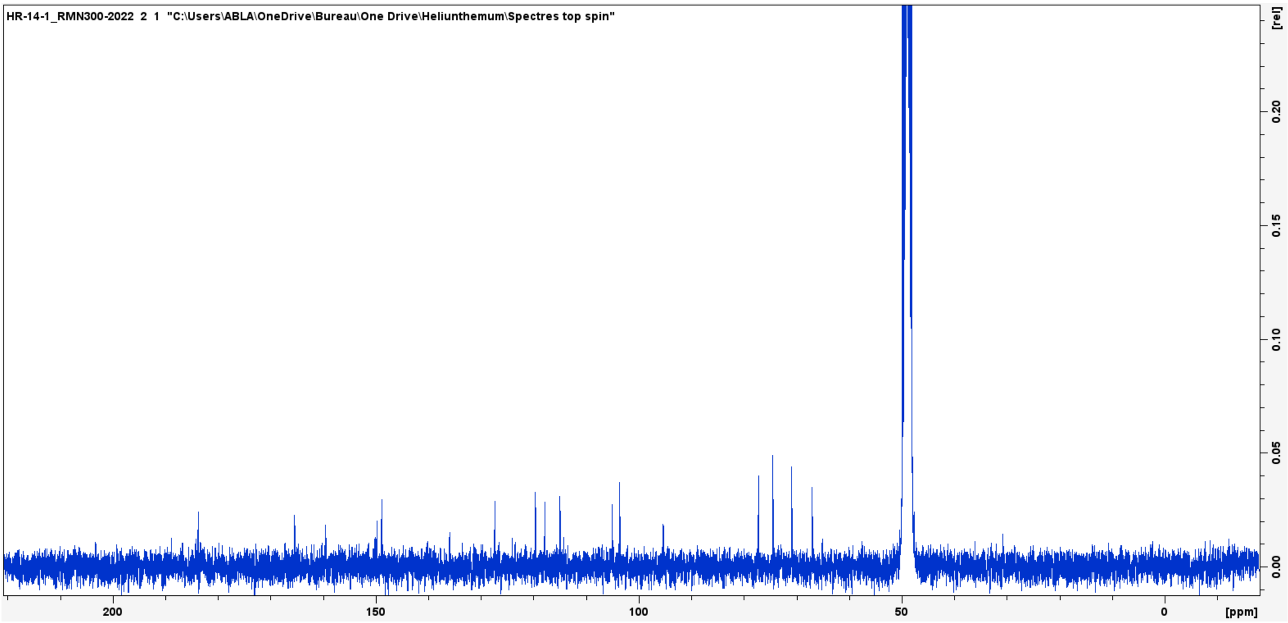

S2. 13C NMR spectrum of compound 3 (75 MHz, CD3OD)

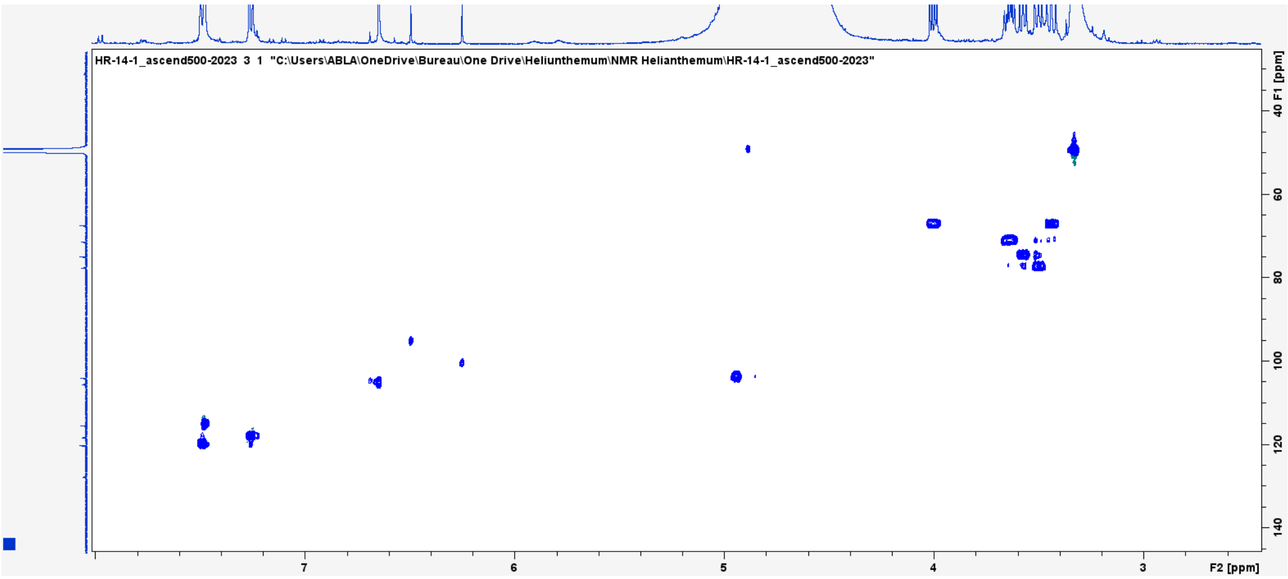

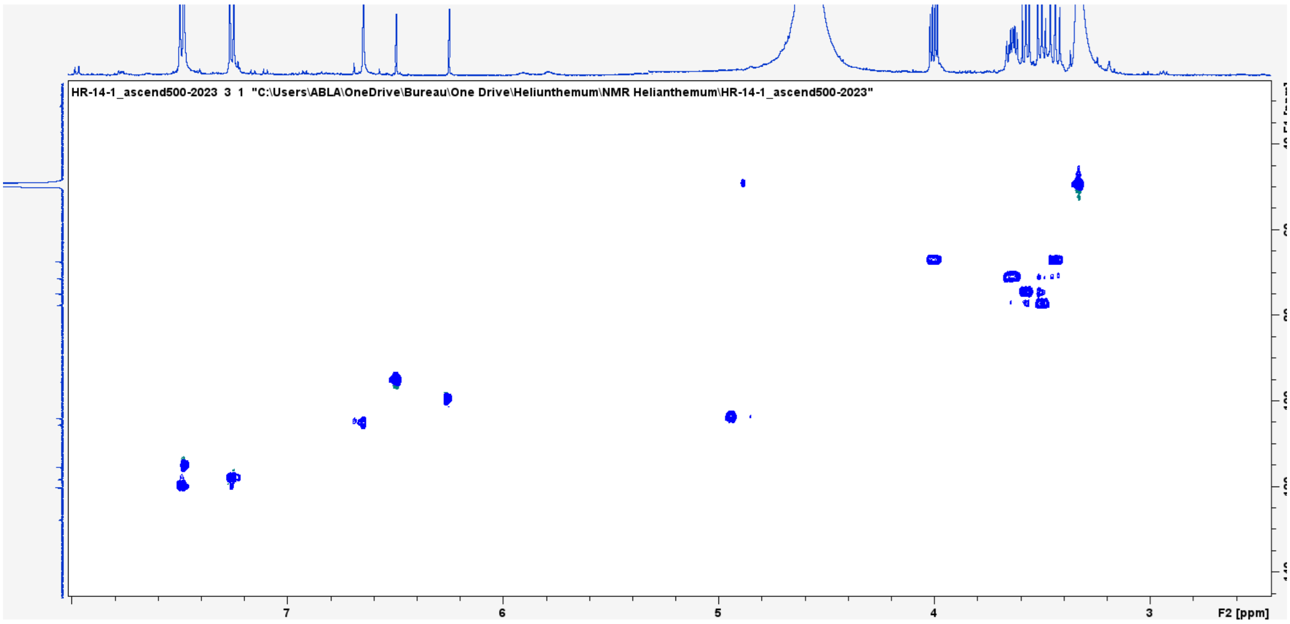

S.3 HSQC spectrum of compound (3) (500 MHz, CD3OD)

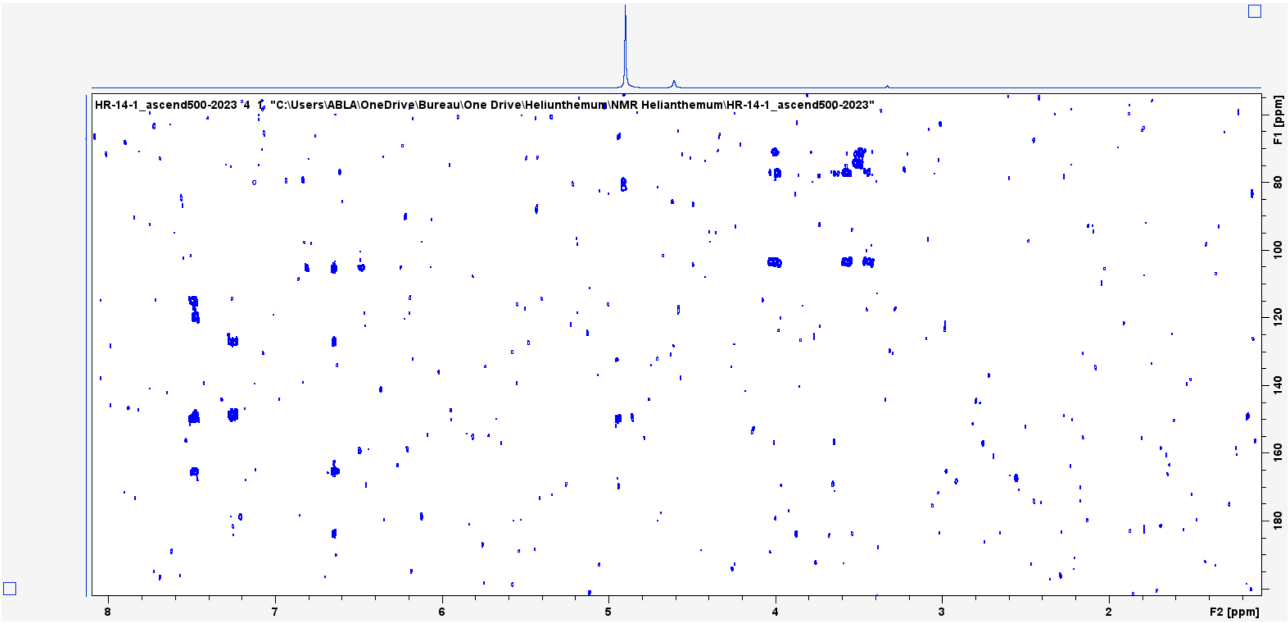

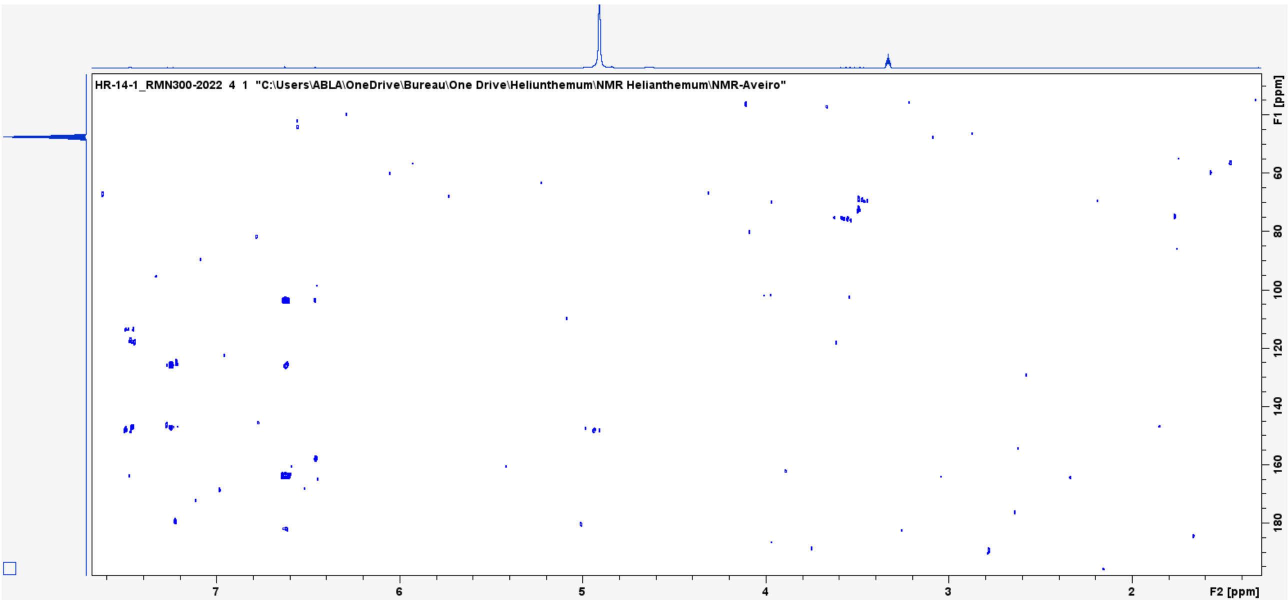

S4. HMBC spectrum of compound (3) (300 MHz, CD3OD)

Compound (4): Luteolin 4'-*O*-β-glucoside

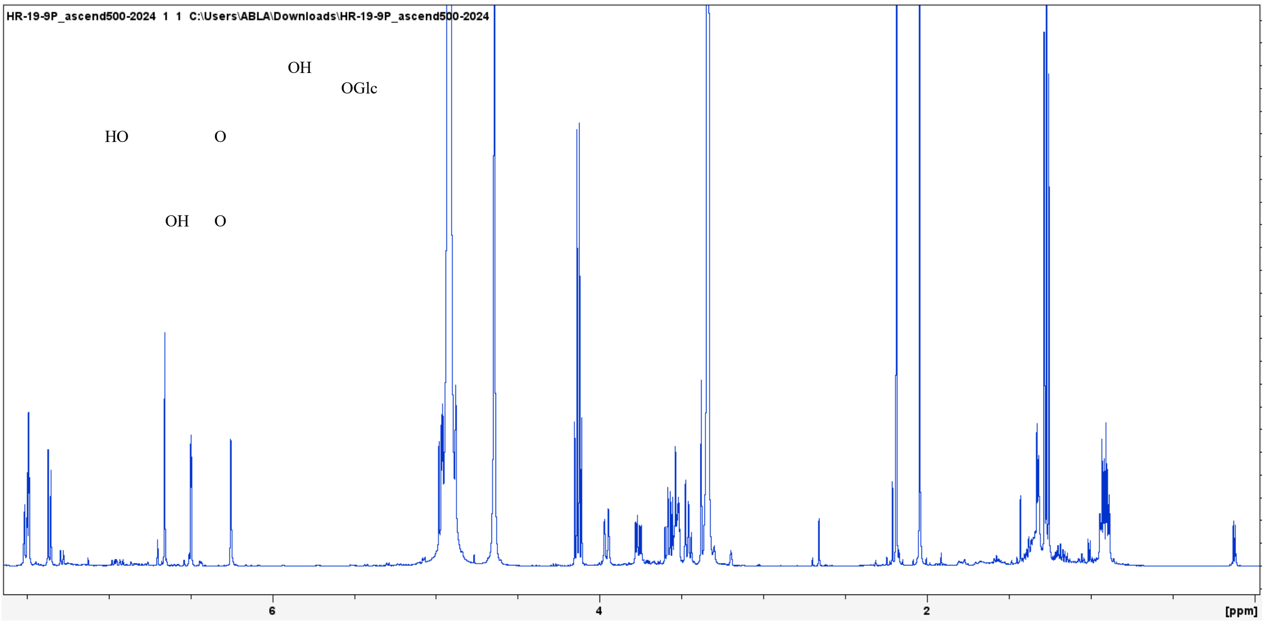

S5. 1H NMR spectrum of compound (4) (500 MHz, CD3OD)

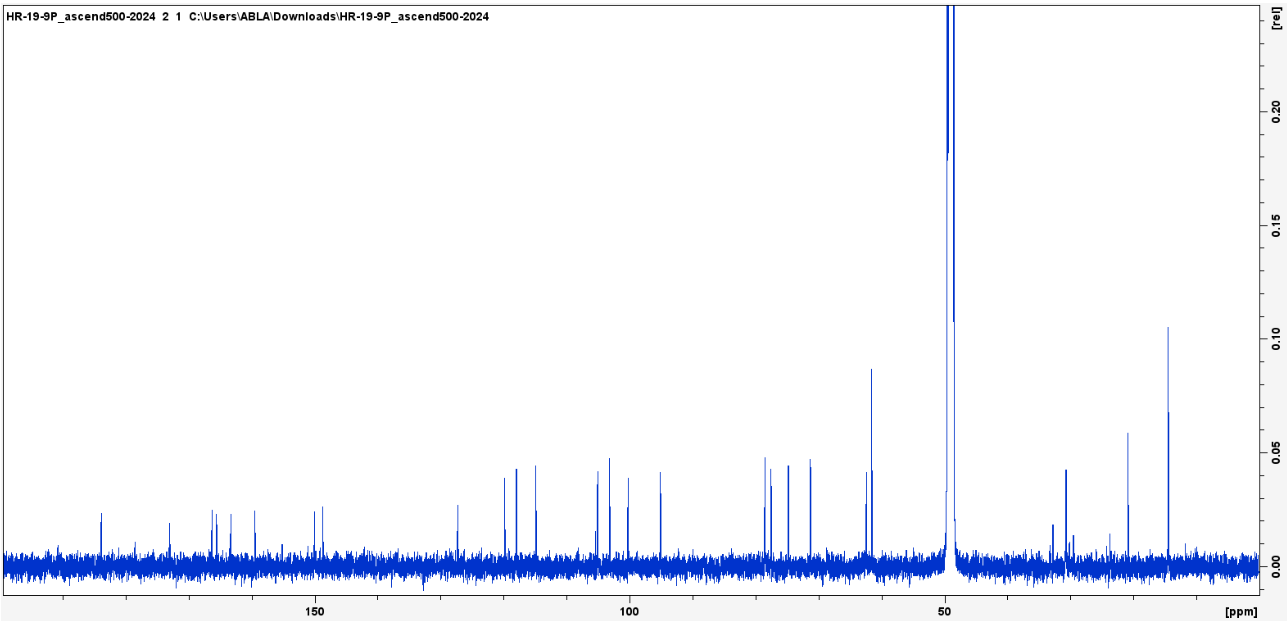

S6. 13C NMR spectrum of compound (4) (125 MHz, CD3OD)

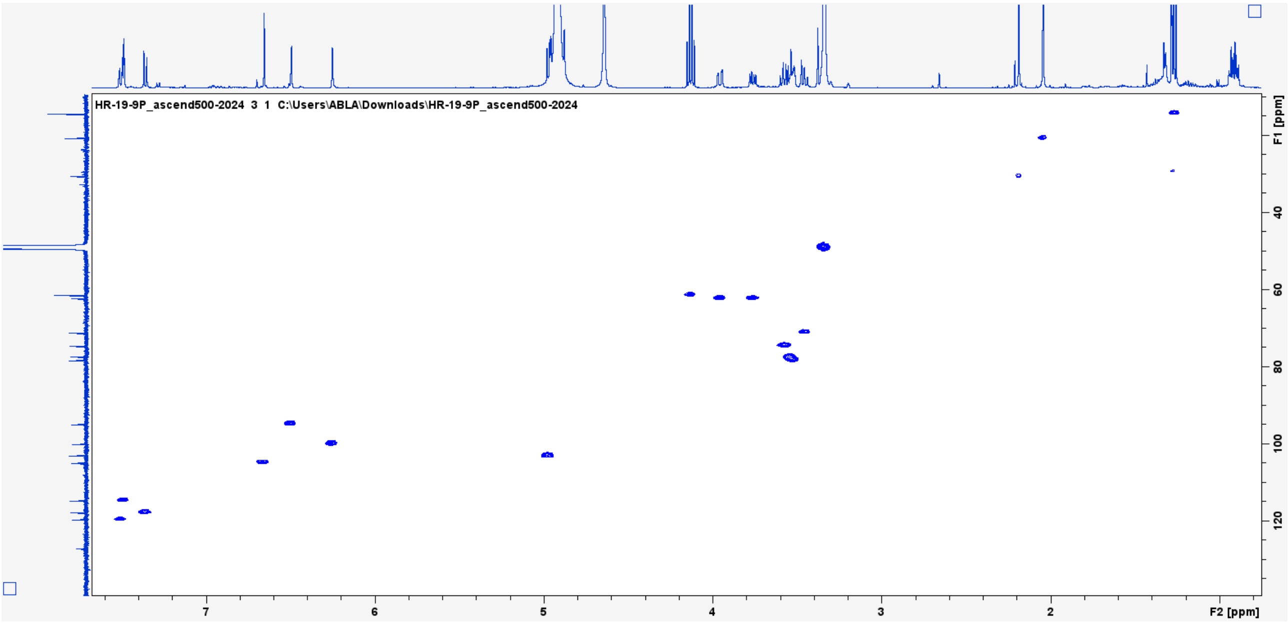

S7. HSQC spectrum of compound (4) (500 MHz, CD3OD)

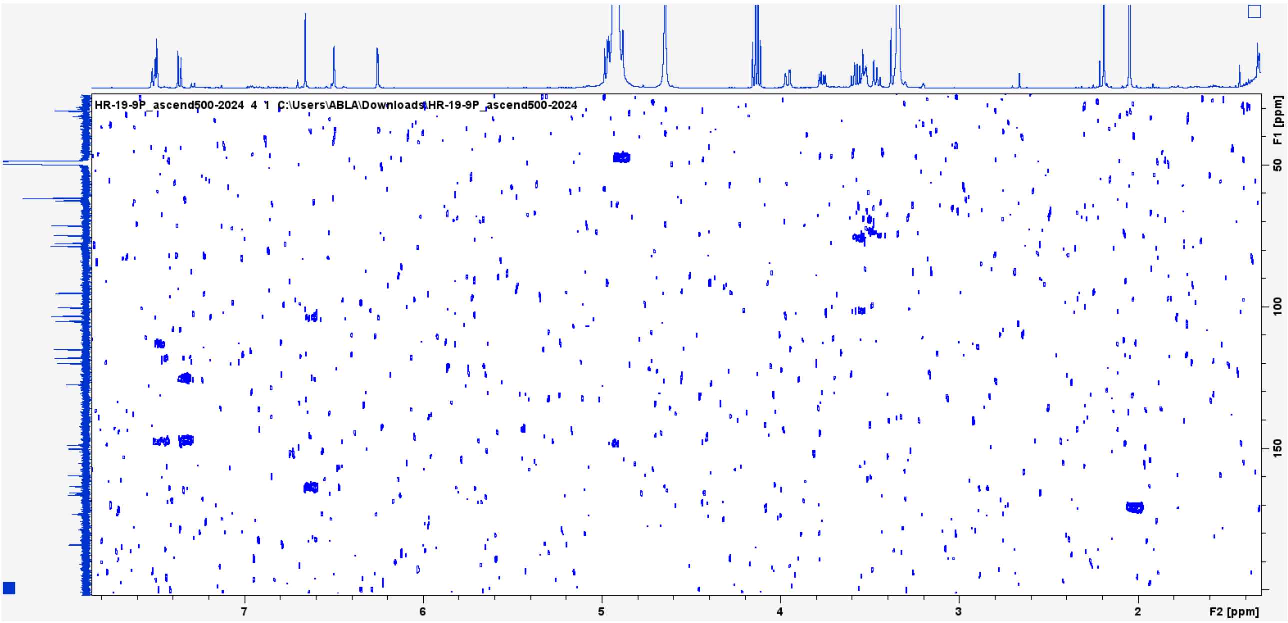

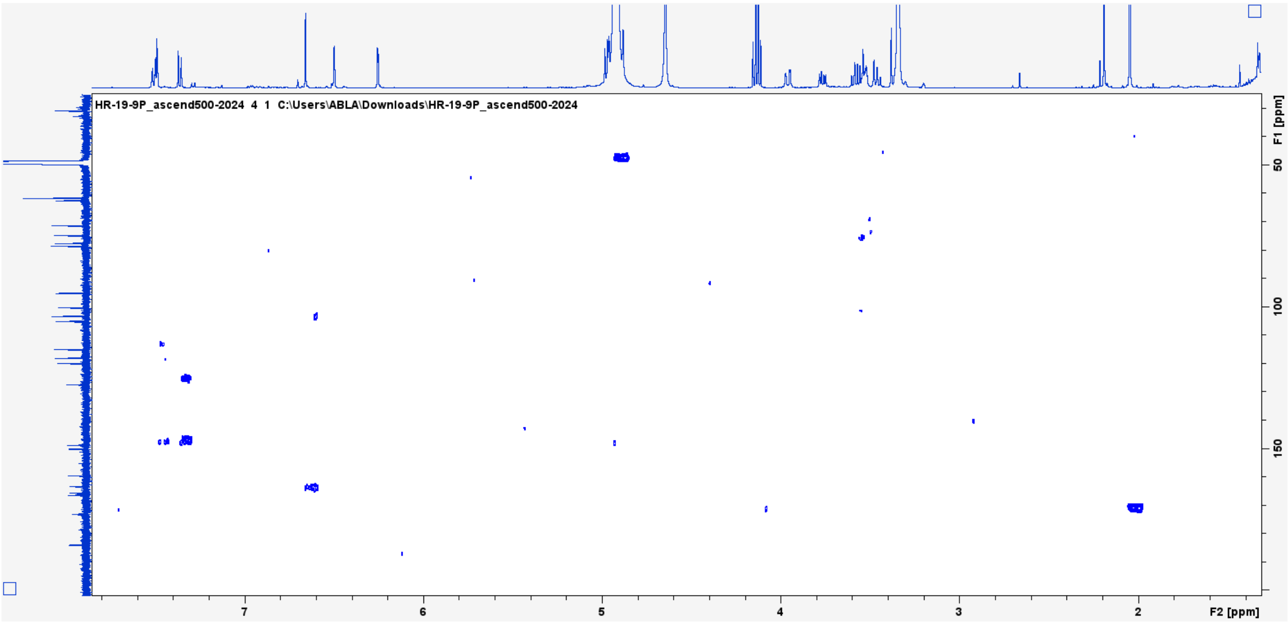

S8. HMBC spectrum of compound (4) (500 MHz, CD3OD)

Compound (5): Quercetin 4'-*O*-β-xyloside

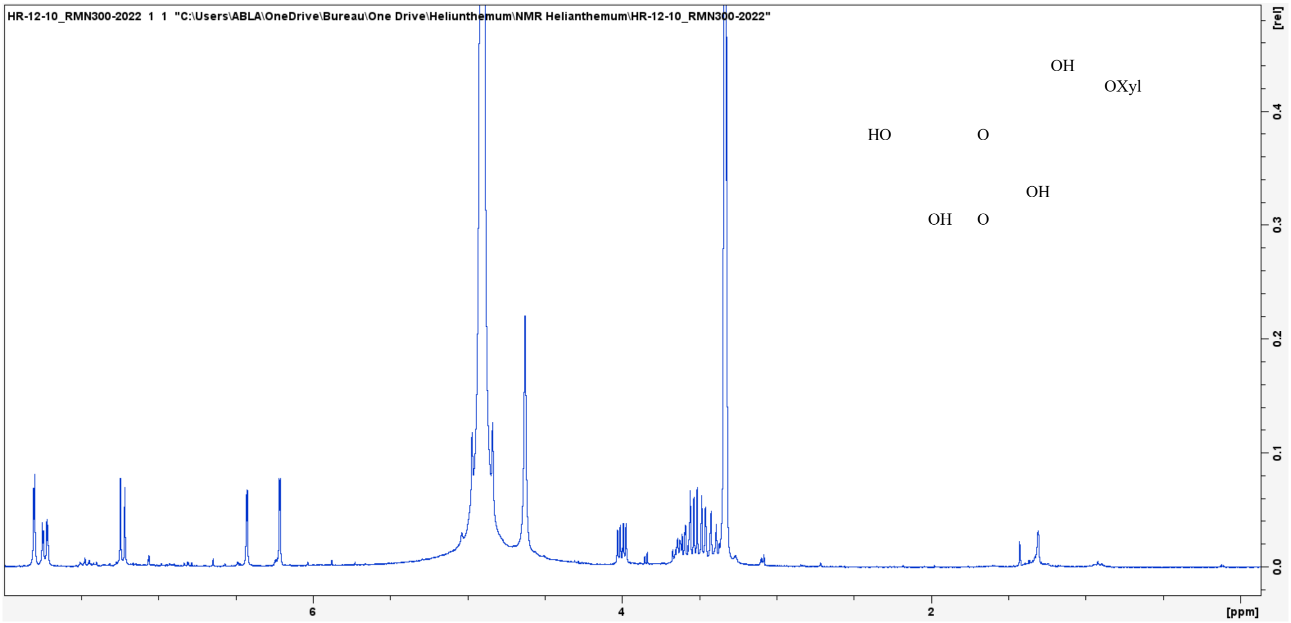

S9. 1H NMR spectrum of compound (1) (300 MHz, MeOD)

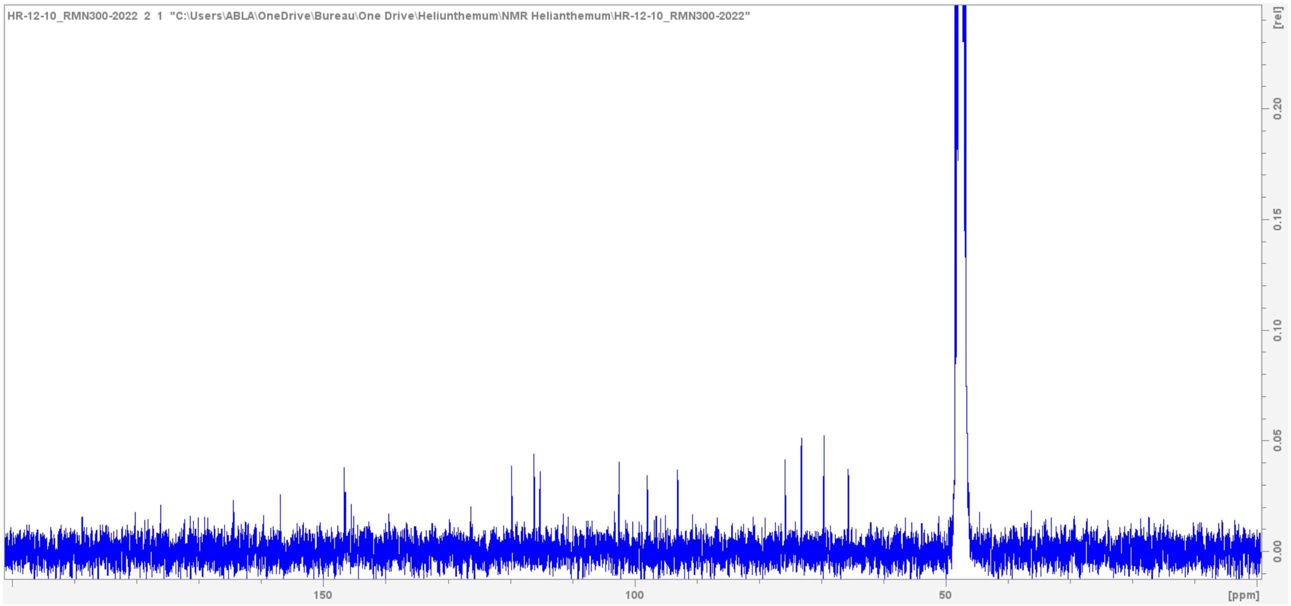

S10. 13C NMR spectrum of compound (4) (75 MHz, CD3OD)

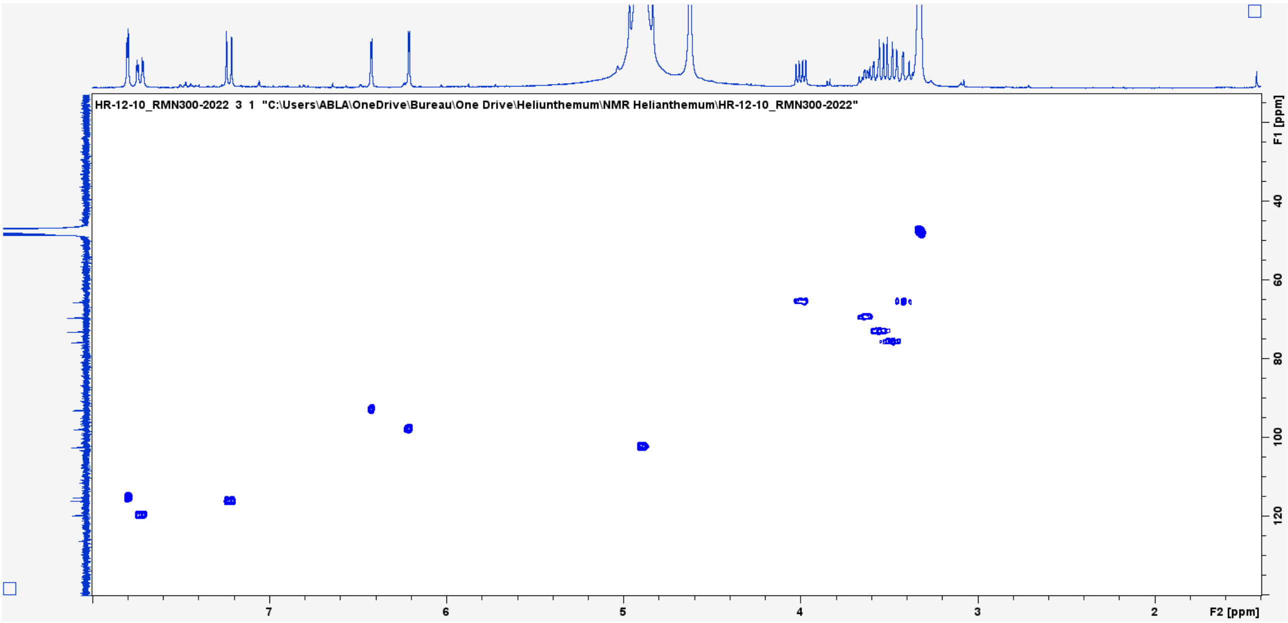

S11. HSQC spectrum of compound (5) (500 MHz, CD3OD)

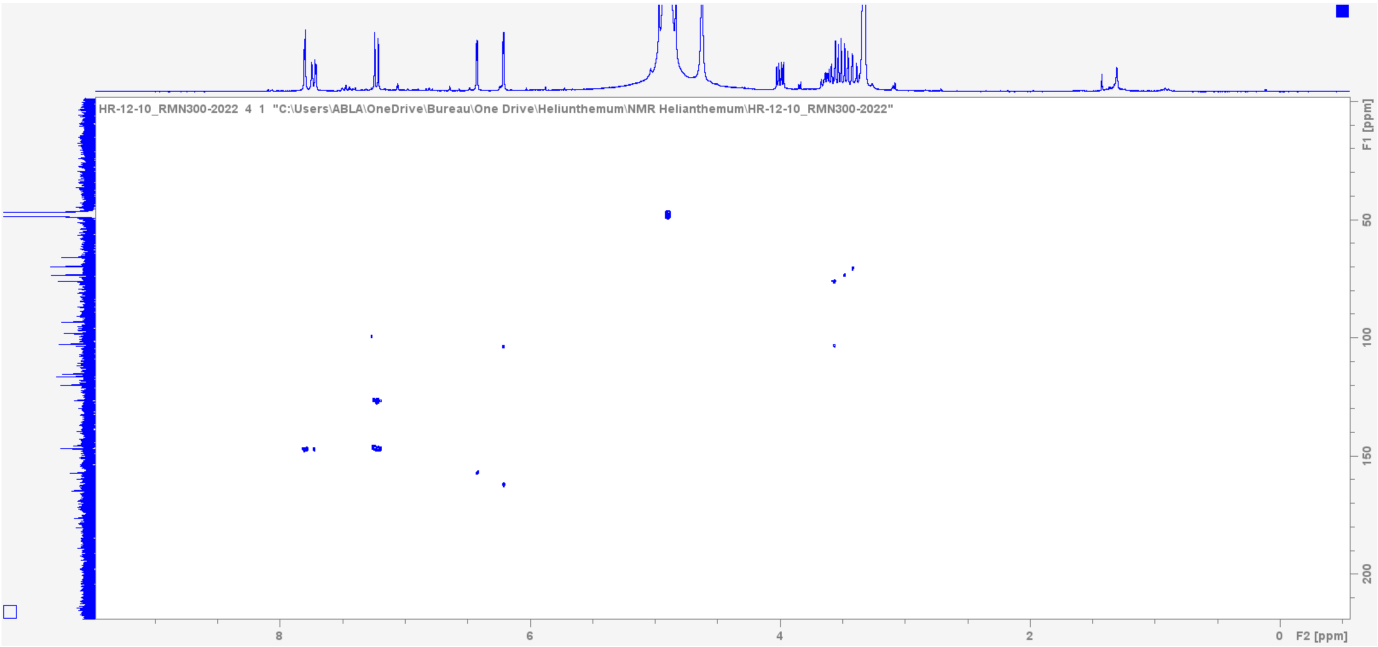

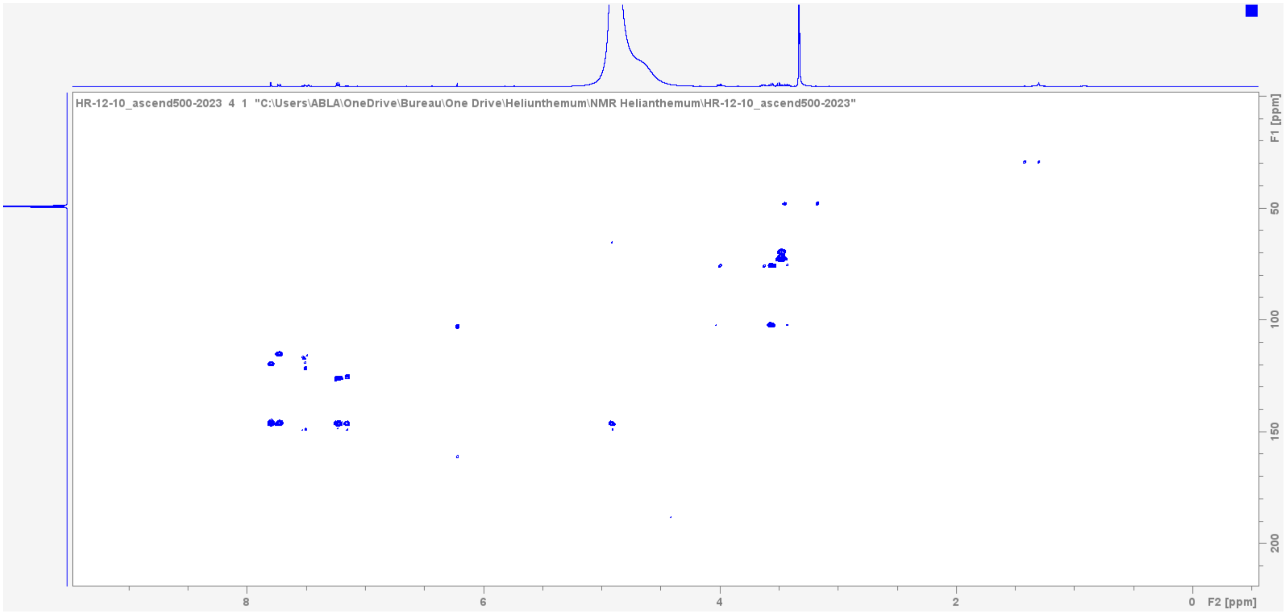

S12. HMBC spectrum of compound (5) (300 MHz, CD3OD)

Compound (6): Kaempferol-3-*O*-[6"-*O*-(*E*)-p-coumaroyl]-β-D-glucoside (*trans*-tiliroside)

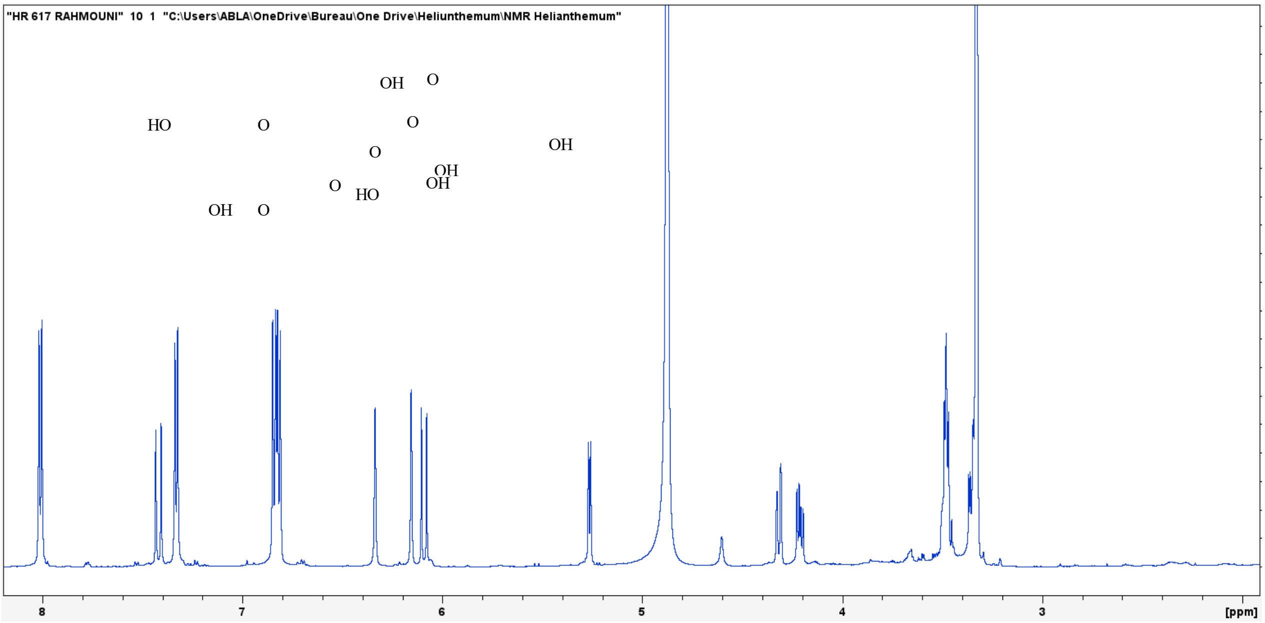

S13. 1H NMR spectrum of compound (6) (600 MHz, CD3OD)

Compound (11): Shikimic acid 3-*O*-gallate

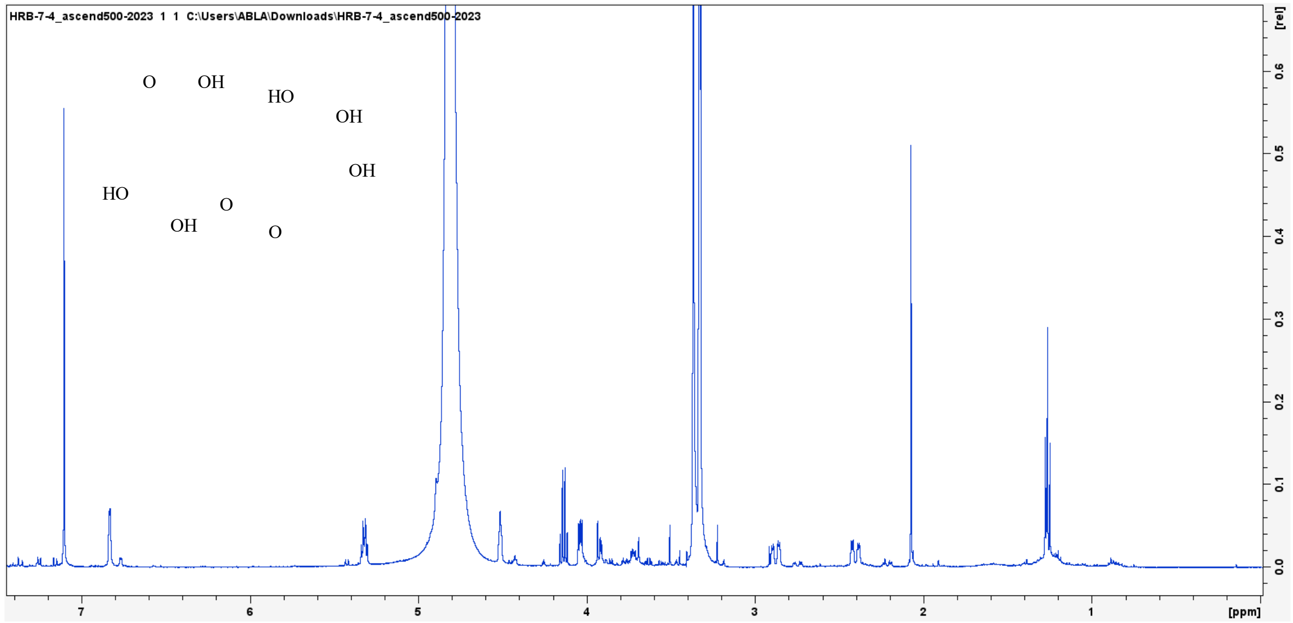

S14. 1H NMR spectrum of compound (11) (500 MHz, CD3OD).

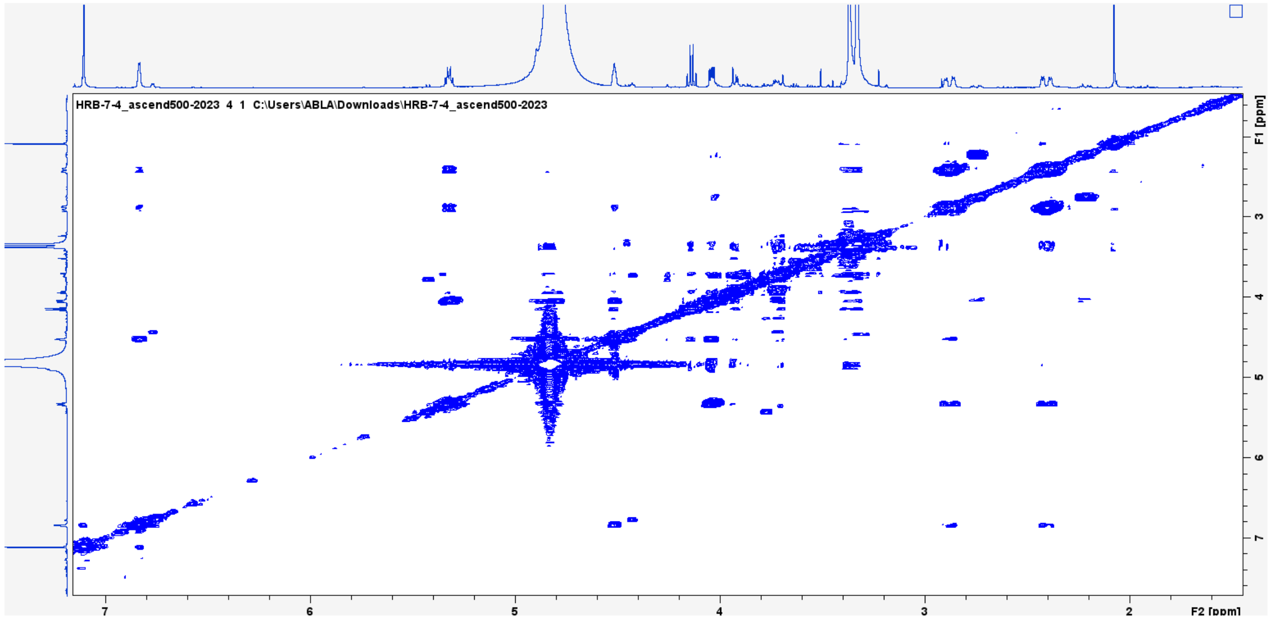

S15. COSY spectrum of compound (11) (500 MHz, CD3OD)

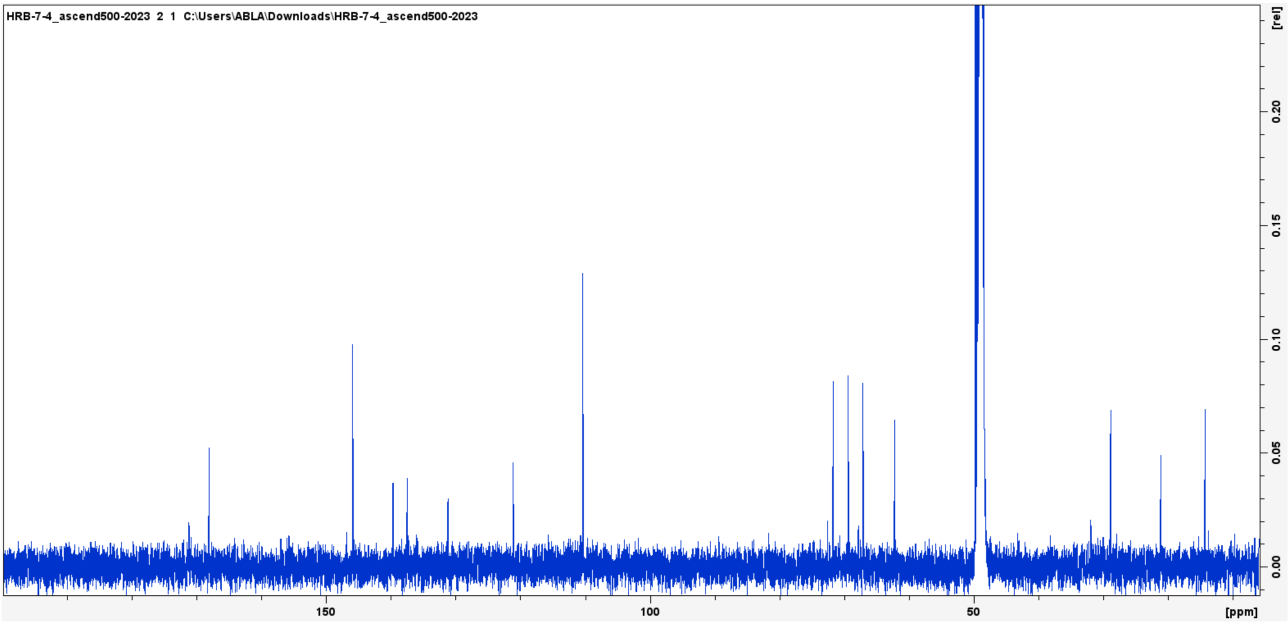

S16. 13C spectrum of compound (11) (500 MHz, CD3OD)

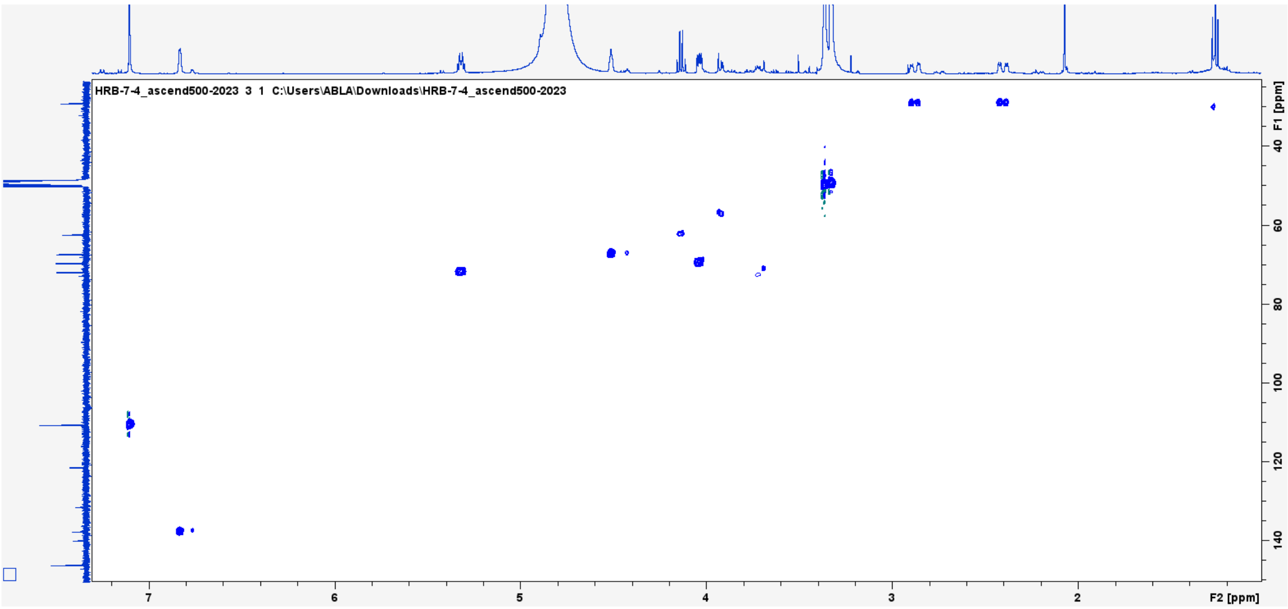

S17.HSQC spectrum of compound (11) (500 MHz, CD3OD)

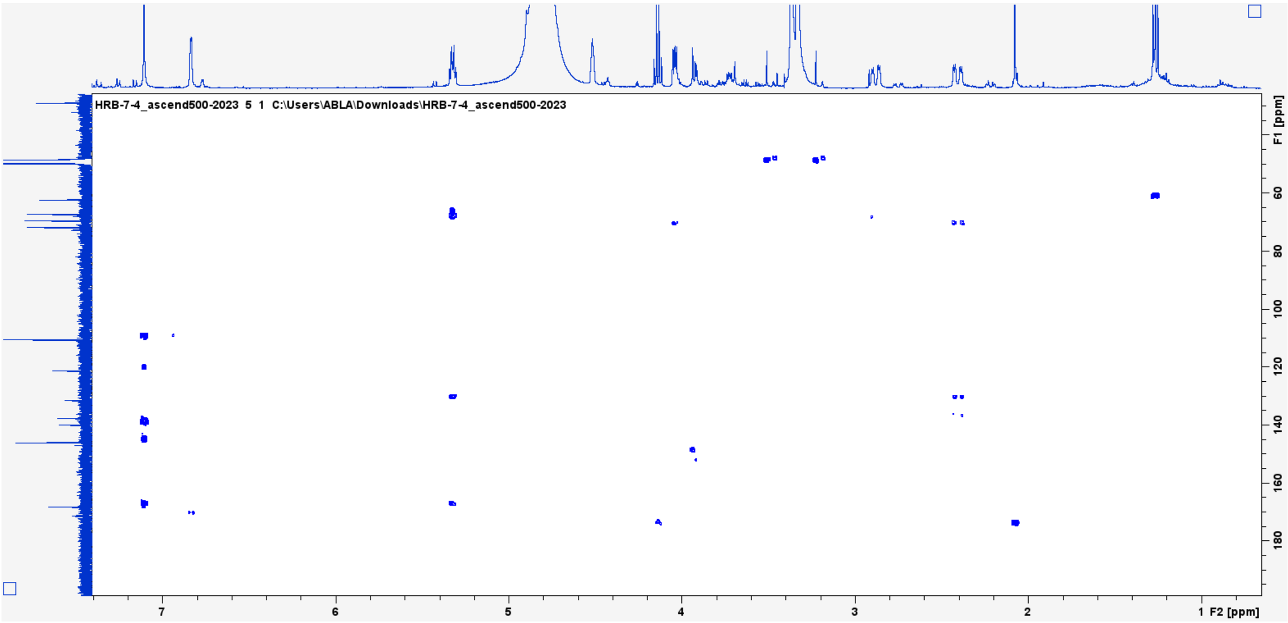

S18. HMBC spectrum of compound (11) (500 MHz, CD3OD)

Compound (12): 3,3',4'-tri-*O*-methyl-ellagic acid 4-sulfate

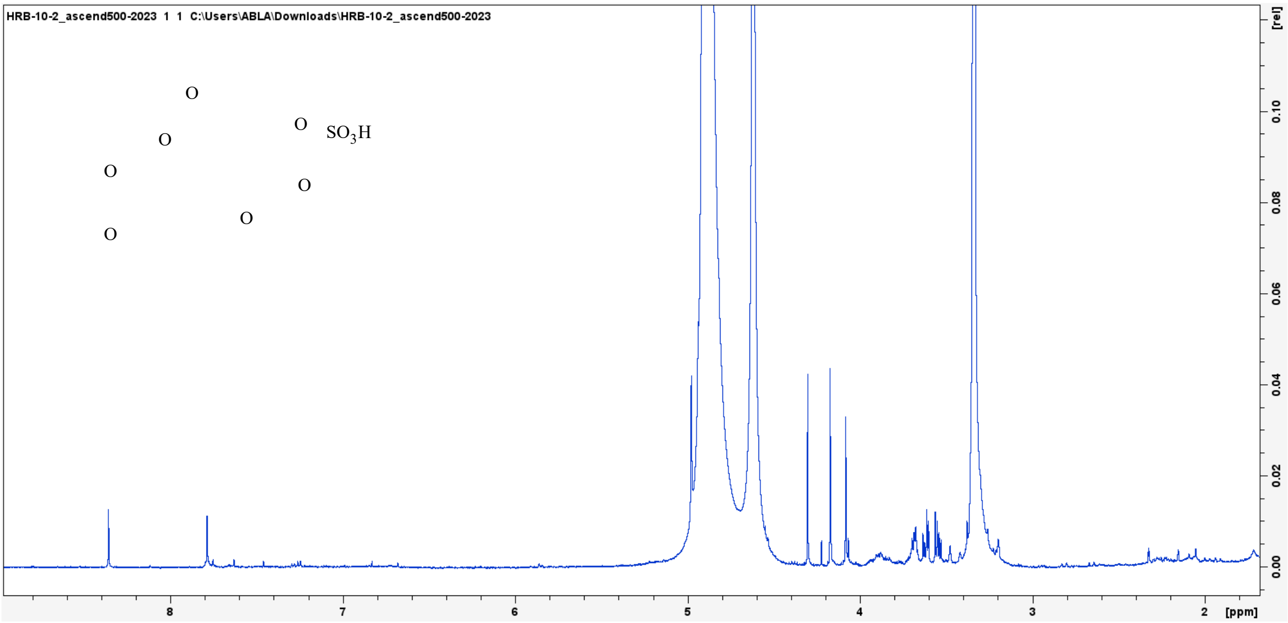

S19. 1H NMR spectrum of compound (12) (500 MHz, CD3OD)

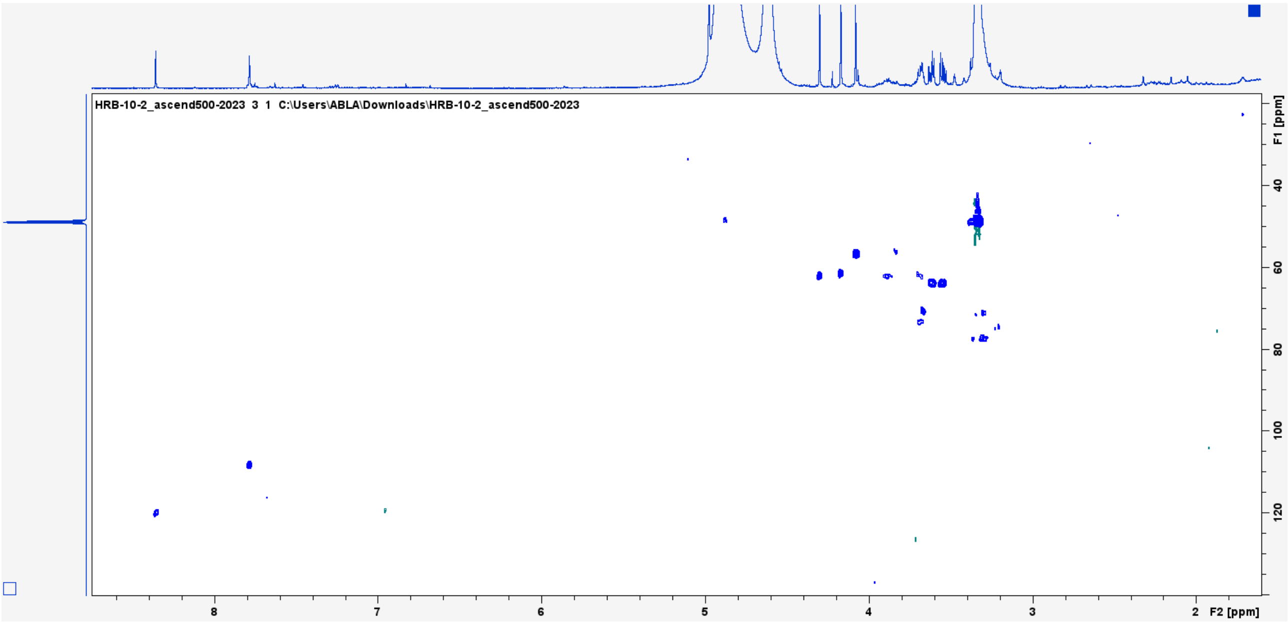

S20. HSQC spectrum of compound (12) (500 MHz, CD3OD)

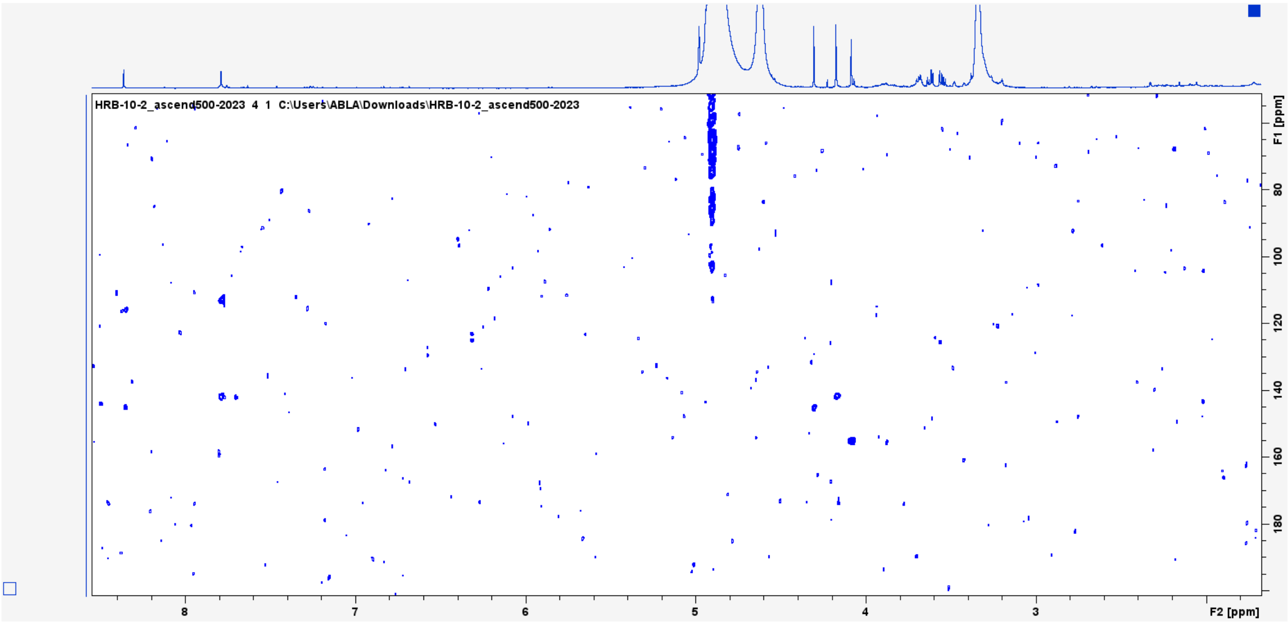

S21. HMBC spectrum of compound (12) (500 MHz, CD3OD)

S1. Mass spectrum of compound (3)

S2. Mass spectrum of compound (4)

S3. Mass spectrum of compound (5)

S4. Mass spectrum of compound (11)

S5. Mass spectrum of compound (12)

Compound (3) : (m/z = 418 uma)

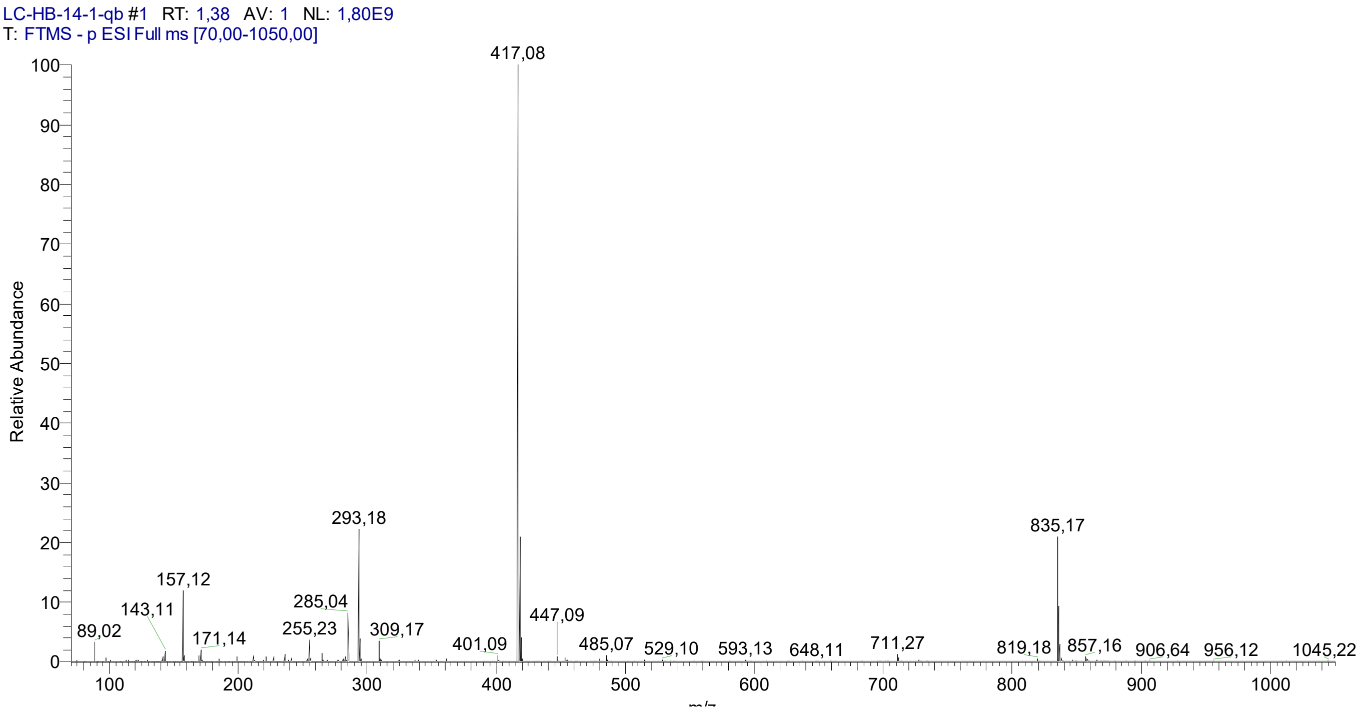

S1. Mass spectrum of compound (3) **$[M - H]^-$ = 417,08**

Compound (4) : (m/z = 448 uma)

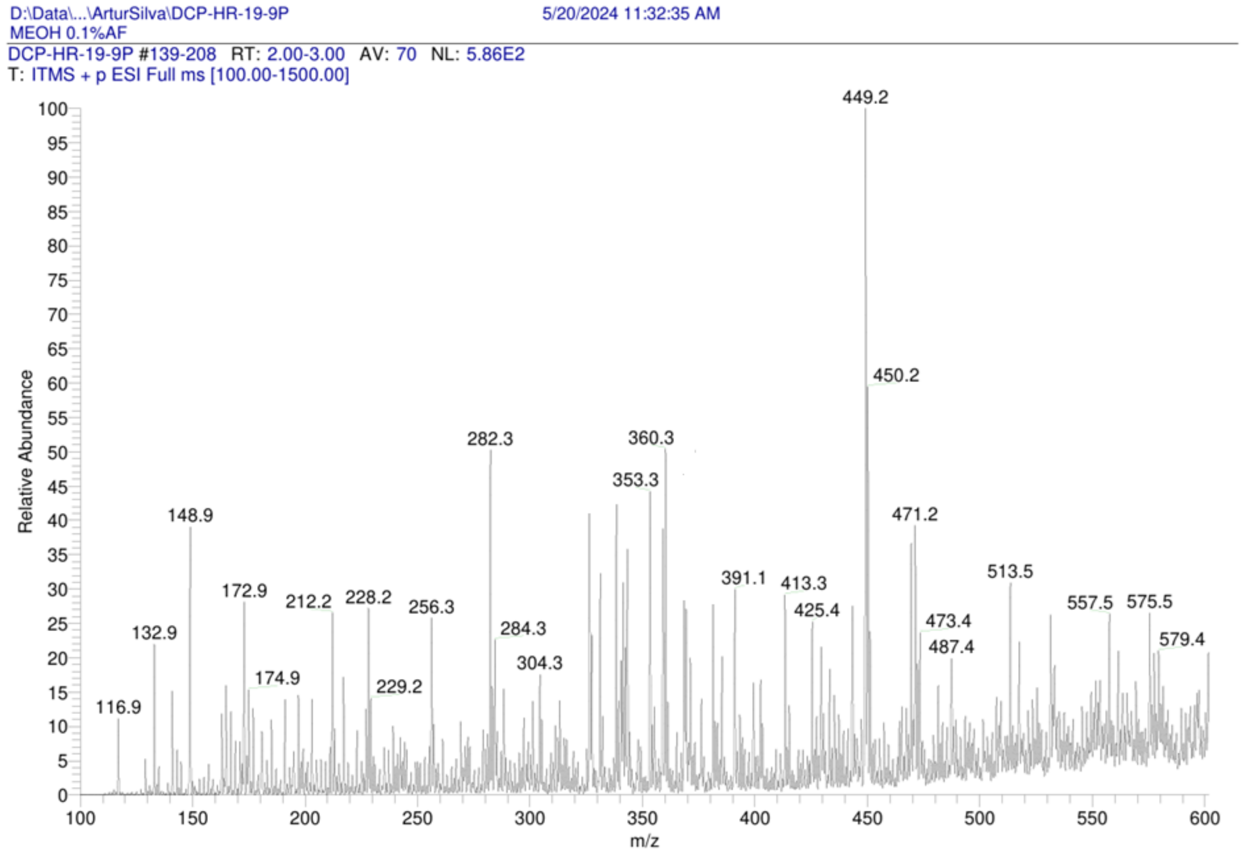

S2. Mass spectrum of compound (4) **$[M + H]^+$ = 449,2**

Compound (5) : m/z = 434 uma

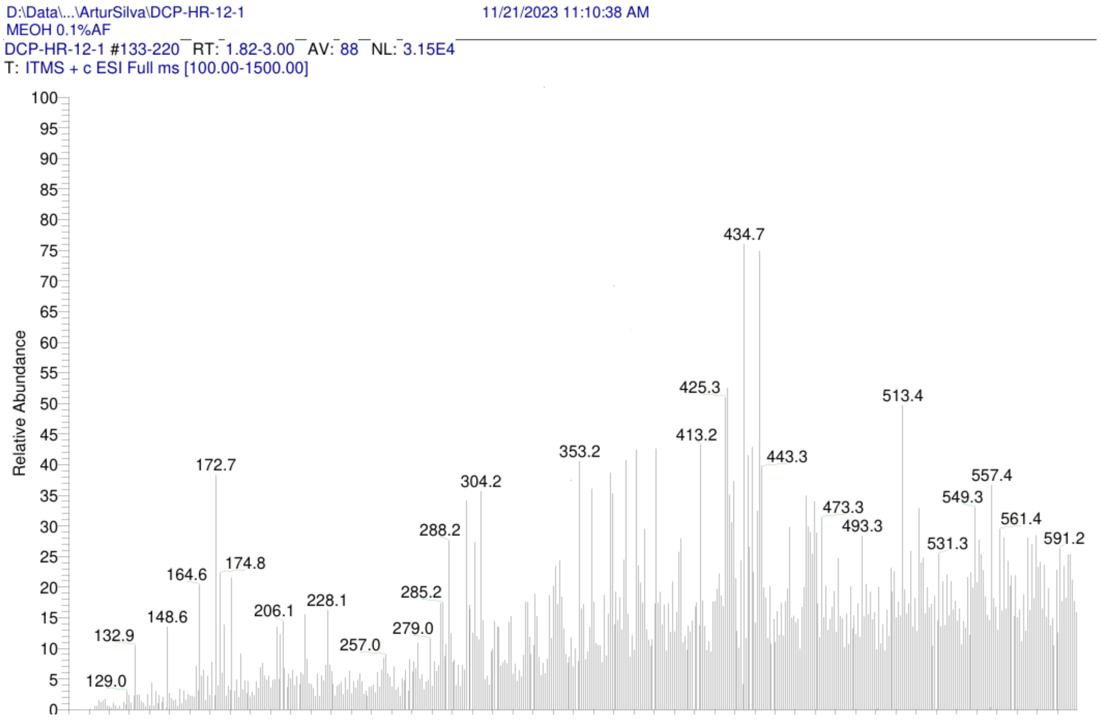

S3. Mass spectrum of compound (5) **[M + H]⁺ = 434,7**

Compound (11) : (m/z=326 uma)

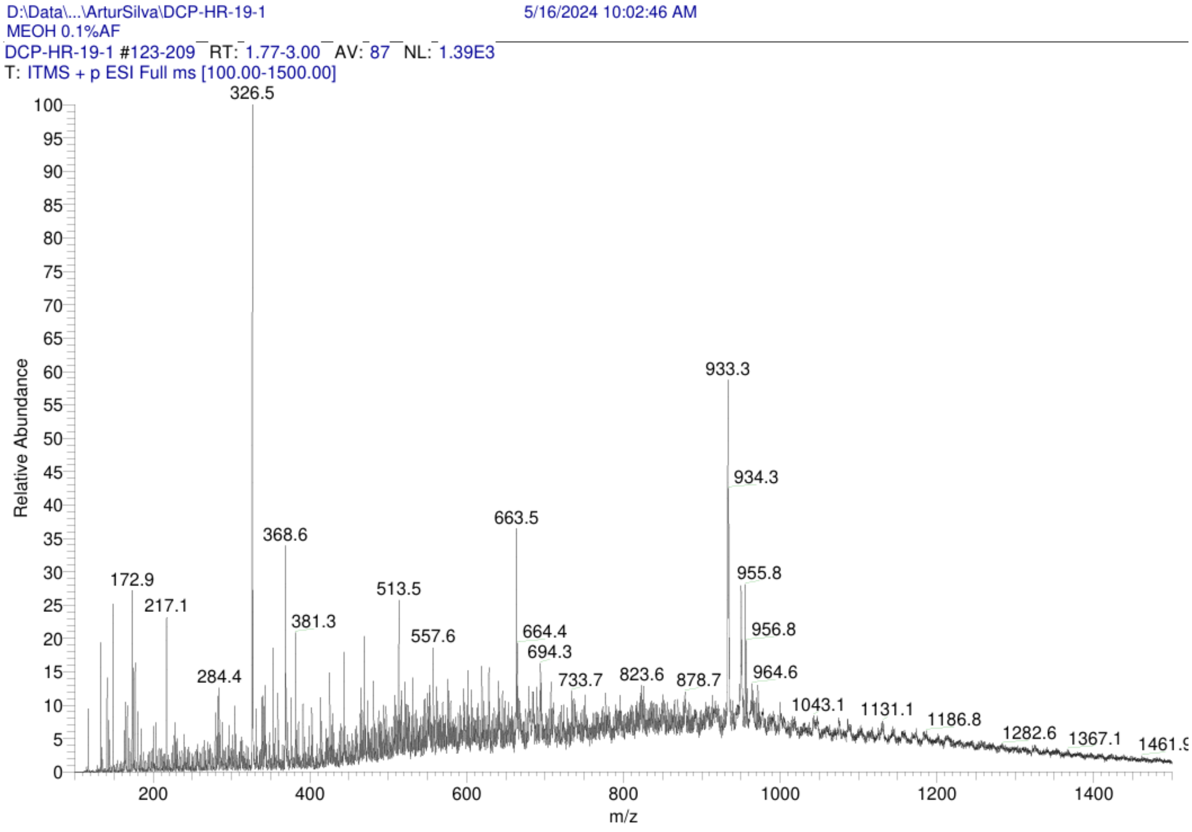

S4. Mass spectrum of compound (11) **[M + H]⁺ = 326,5**

Compound (12) : (m/z=424 uma)

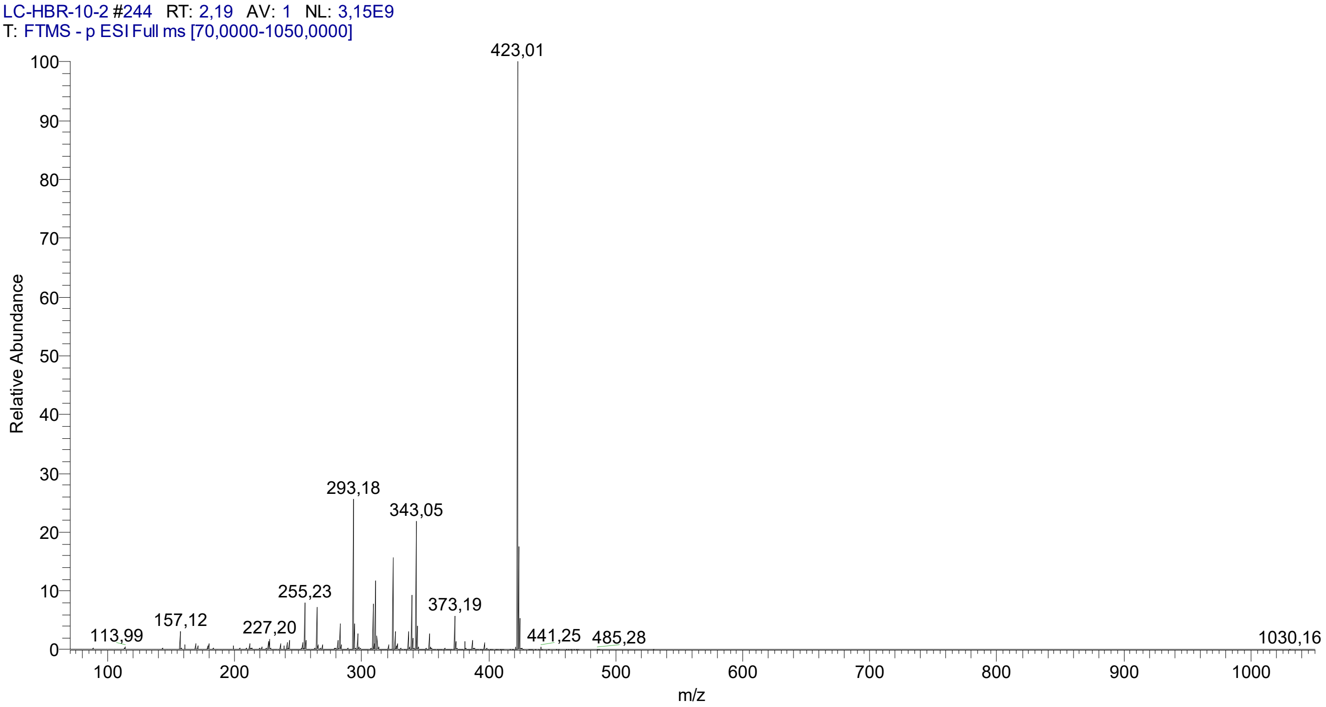

S5. Mass spectrum of compound (12) **[M - H]$^{-}$ = 423,01**
